# Feasibility of Video Consultation for Preterm Neurodevelopmental Follow-up Care During the COVID-19 Pandemic: Cohort Study

**DOI:** 10.2196/40940

**Published:** 2023-01-25

**Authors:** Bilge Albayrak, Larissa Jane Cordier, Sandra Greve, Uta Teschler, Anne-Kathrin Dathe, Ursula Felderhoff-Müser, Britta Maria Hüning

**Affiliations:** 1 Department of Pediatrics I, Neonatology, Pediatric Intensive Care and Pediatric Neurology University Hospital Essen University of Duisburg-Essen Essen Germany; 2 Center for Translational Neuro- and Behavioral Sciences University Hospital Essen University of Duisburg-Essen Essen Germany

**Keywords:** COVID-19, very preterm infant, video consultation, follow-up care, COVID-19 pandemic, neurodevelopmental outcome

## Abstract

**Background:**

During the COVID-19 pandemic, parents of infants born very preterm or at risk were exceptionally worried about being infected. The only means of protection during the onset of the pandemic was social distancing. Video consultations for neurodevelopmental follow-up care were offered as an alternative way to stay in contact with patients and their families, to provide expert support, and to monitor and assess children’s development.

**Objective:**

To assess the feasibility of and family satisfaction with video consultations, interviews were conducted after video and in-person consultations.

**Methods:**

An interview with 28 questions was created to evaluate parental satisfaction with the consultations (eg, their confidentiality and the children’s behavior). A total of 93 interviews with parents were conducted between March 2020 and February 2021 and compared (58 after video consultations and 35 after in-person consultations). The interviews were conducted at the end of the consultations by a trained professional. The video consultations were conducted using a certified platform created by Zava Sprechstunde Online, maintaining data protection with end-to-end encryption. Follow-up consultations (video or in-person) were performed at corrected ages of 3, 6, and 12 months as well as 2, 3, 4, and 5 years. The rate of total follow-up appointments attended during the survey period was evaluated and compared with the previous year.

**Results:**

There were no significant differences between the video and in-person consultation groups in satisfaction, attitudes on the confidentiality of the consultation, or discussion of private and sensitive information. Following video consultations, parents were significantly more likely to report that they were avoiding contact with medical professionals during the pandemic (*P*=.045; Shapiro-Wilk *W*=1094.5, Cohen *d*=–0.1782146) than the in-person consultation group. Parents in the video-consultation group stated that performing a guided examination on their child was comfortable and helped them understand their child’s development. In fact, they agreed to take advantage of future video consultations. The rate of total follow-up appointments increased compared to the previous year. Between March 2019 and February 2020, 782 of 984 (79.5%) children born at Essen University Hospital attended a follow-up appointment. During the survey period, between March 2020 and February 2021, a total of 788 of 1086 children (73%) attended a follow-up appointment, of which 117 (14.9%) were video consultations.

**Conclusions:**

The feasibility of attending video consultations for follow-up care of very preterm or at-risk infants and parental satisfaction with these consultations were as high as for in-person consultations. Parents rated video consultations as being as confidential as in-person appointments. Telemedicine can be offered as an equivalent alternative to in-person consultations and is particularly useful under certain circumstances, such as for very sick children who require assistive devices or respiratory support and oxygen or for those living a long distance away.

## Introduction

Approximately 15 million newborns are born preterm worldwide each year, which is more than 1 in 10, and this rate is increasing in almost every country. Substantial progress in perinatal care has significantly increased survival rates over the past 3 decades. However, long-term morbidity remains a concern [1,2]. Approximately 14% of newborn infants (preterm and term-born infants) need postnatal care in Germany, and 8.6% of infants are born preterm [3,4]. Therefore, follow-up care for these patients at risk is an important part of a safe transition from the neonatal intensive care unit (NICU) to home care, and it is crucial for long-term monitoring and support of these infants’ neurodevelopment [5]. For early detection and intervention, international guidelines recommend follow-up examinations at defined times, starting with discharge management and support during the transition to school age [6]. The use of standardized tests is advisable, as well as assessment of the physical and mental health of both children and parents. Other important aspects of follow-up consultations are to support parent-child interactions, provide information on how parents can promote their child’s development, and give practical recommendations about the challenges of daily life, such as feeding, sleeping, dealing with behavioral problems, and monitoring and administering drugs [5,7].

Whereas follow-up care in very preterm children can detect severe motor and cognitive impairment for up to 2 years, long-term follow-up care, until school age, is recommended to detect more subtle development disorders [8]. Regular follow-up appointments enable early intervention in children who have previously demonstrated age-appropriate development, but later show development disturbances. However, during the COVID-19 pandemic, appointments may have been missed at critical periods of development. The reasons for this are manifold: symptoms of infection, quarantine and hygiene restrictions, and fear of infection.

At the onset of the COVID-19 pandemic, neither vaccination nor adequate therapies were available, and the only means of protection was social distancing. To reach out to patients and their families who would not or could not attend regular follow-up appointments, video consultations were offered. This method provided several opportunities: it allowed health care providers to stay in contact with patients and their families, provide expert support and information, and keep track of the children’s development. However, there were potential challenges, such as a lack of confidentiality, the discussion of private and sensitive information, technical issues, accessibility, and acceptance of this new digital tool. To assess the feasibility of this method and family satisfaction, we conducted interviews after video and in-person consultations. The rate of total follow-up appointments attended during the period of the survey, which took place during the COVID-19 pandemic, was evaluated and compared with the previous year.

## Methods

### Subjects

A total of 93 interviews were conducted with parents (58 after a video consultation and 35 after an in-person outpatient appointment) between March 2020 and February 2021. On average, parents of the participants were aged 33.45 (SD 5.11) years in the video-consultation group and 31.40 (SD 5.86) years in the in-person group. Inclusion criteria for participation in the video-consultation group were sufficient language skills in German, English, or Turkish and the possession of the minimum required technical equipment, such as a mobile phone. Interviews were conducted at the end of the in-person or digital appointment by a professionally trained neurodevelopmental psychologist. The video consultations were conducted on a certified platform developed by Zava Sprechstunde Online that maintained data protection with end-to-end encryption.

### Ethics Approval

The investigation was approved by the ethics committee of the Medical Faculty of the University Hospital Essen (20-9319-BO) and adhered to the Declaration of Helsinki.

### Planning and Implementation of Digital Consultations at the Onset of the COVID-19 Pandemic

Starting on March 13, 2020, the German federal states ordered the closure of schools and kindergartens, postponed semester breaks, and banned visits to nursing homes to protect the elderly [9]. Two days later, the borders with Austria, Denmark, France, Luxembourg, and Switzerland were closed. By March 22, curfews had been imposed in 6 states, while others prohibited physical contact with more than one person outside the household [10]. To protect the most fragile patients, such as follow-up groups of premature or high-risk infants, the department of neurodevelopmental follow-up care at our institution sought an alternative as soon as possible to protect patients and their families from the unknown COVID-19 virus. All high-risk patients and families already scheduled for in-person appointments were contacted and asked if they would be willing to change to a video consultation. If they declined, the in-person appointment was performed wearing protective equipment and was scheduled so that patients did not meet each other. If a family agreed to hold the scheduled appointment as a video consultation, they received an email in advance with the necessary technical requirements and items to have ready for the appointment (eg, an ID card to identify themselves, an internet-enabled device with a camera, and toys). After the video consultation and outpatient appointment with the staff, an interview (described in the Measurements section of this paper) was conducted with the same staff member to compare the quality of the appointment to an in-person consultation. This was highly important to the department of neurodevelopmental follow-up care, as it was crucial to know whether digital appointments via video consultation were feasible during this time of uncertainty in the pandemic. The participants in the video interviews could decide whether they wanted to take part or not. They had the option to cancel the interview and recall their data at any time. Due to the completely anonymous collection of the data, no conclusions can be drawn about the families, the age of the patients, or whether the same families participated in multiple video consultations.

### Video Consultation Procedure

Follow-up care, both via video consultation and in person, was performed by authors BMH, BA, and SG (who are medical doctors), LJC (a psychologist) AKD (an occupational therapist) and UT (a physiotherapist). During the in-person visits, a detailed medical history was taken with the parents, and the child was then examined by the various professionals. During video consultations, a detailed medical history was also taken, but the camera was pointed at the child so that he or she could be observed in his or her home environment. After the medical history was taken, parents were subsequently instructed to position the child and the camera. The parents were guided with a mannequin through different positions of the infant, such as a traction attempt, held seat and held stand, the axillary hanging position, the floating stomach position, and the passive rotation and stomach position. The spontaneous movements of the infant in these different positions were observed. Preschool-age children were instructed to perform fine and visual motor tasks, including copying forms; using scissors to cut along a line; and grasping, transferring, and releasing paper clips into a match box, to test precise finger coordination. Gross motor functions were tested using regular neurological examinations, such as the finger-nose test, walking in tandem, and the stance and gait test.

While guiding parents, the examiner explained all observed items and findings. In addition, questions regarding the examination, as well as everyday life issues, were answered, including questions related to weight gain, feeding, developmental steps, and self-regulation. Physicians were able to assess fine and gross motor skills, as well as cognitive function, in the children through visual observation, for example, by observing how a toddler explored and handled items.

### Measurements

To determine the parents’ perspectives on video and in-person consultations at the follow-up outpatient clinic for preterm and high-risk infants during the COVID-19 pandemic, an interview with 26 questions was created based on a questionnaire by Rutherford et al [11] (Multimedia Appendix 1). Questions 1 to 5 related to demographic information and distance from the outpatient clinic. Questions 6 to 20 (for the in-person appointment interviews) and 6 to 26 (for the video consultation interviews) related to parents’ satisfaction with the appointments. Parents’ satisfaction was reported on a Likert scale ranging from 1 (strongly disagree) to 7 (strongly agree). The 6 additional questions in the interviews after the video consultations were related to technical implementation and feasibility (eg, the kind of device used, satisfaction with the video and audio quality, self-examination of the child, instructions on position changes and explanations of development, differences with in-person appointments, and likelihood to use future video consultation).

To evaluate the total attendance rates for follow-up appointments (for both in-person appointments and video consultations) for the children, the total number of consultations was compared with the number of scheduled appointments for preterm infants born at University Hospital Essen and also compared with the previous year.

### Data Analysis

Statistical analyses were conducted using the R environment for statistical computing and graphics with the RStudio integrated development environment. All data were tested for normal distribution using the Shapiro-Wilk test. The distribution was not normal, so all comparisons between groups (ie, the video consultation and in-person appointment groups) were calculated using the Mann-Whitney *U* test. In cases with significant differences, the effect size was calculated with the Cohen *d*.

## Results

Between March 2020 and February 2021, 93 interviews were conducted and analyzed. There were no significant differences between the video and in-person consultation groups in satisfaction or attitudes toward confidentiality and discussion of private and sensitive information. Parents in the video-consultation group were significantly more likely to agree (mean Likert score 4.82, SD 2.26) that they were avoiding contact with medical professionals during the pandemic than the in-person consultation group (mean score 3.89, SD 2.25; Shapiro-Wilk *W*=1094.5; *P*=.045; *d*=–0.1782146). Furthermore, parents in the video-consultation group agreed that they would take advantage of future video consultations (mean score 6.49, SD 1.09). Parents reported being empowered by the self-examination of their child (mean score 6.53. SD 1.39) and learning more about their neurodevelopment. All children were calm and cooperative (mean score 6.45, SD 1.17) in their home setting with only familiar people.

**Figure 1:**
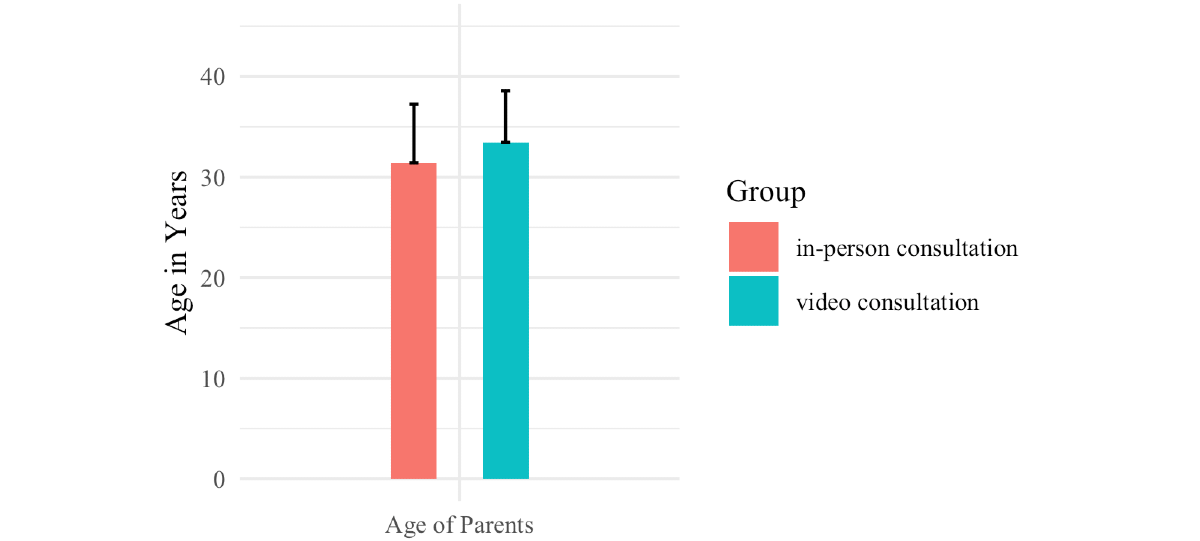
Mean age in years of the parents (*P*=.044 between groups). The bars indicate mean values and tails indicate SD.

**Figure 2:**
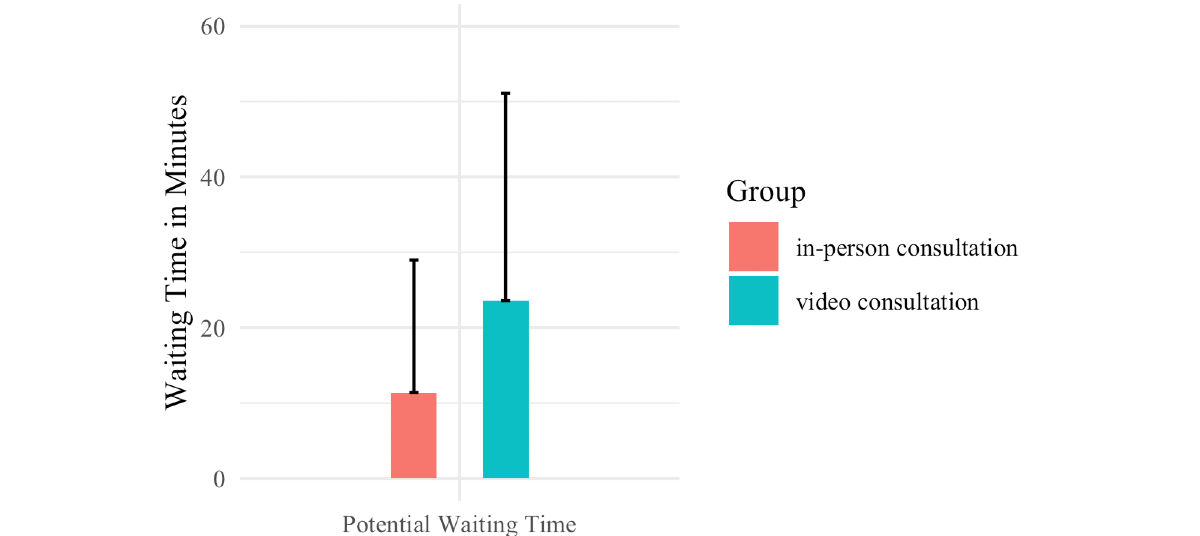
Mean estimates of potential waiting time in minutes for in-person appointments (*P*<.001 between groups). These estimates were made after either an in-person consultation or a video consultation. The bars indicate mean values and tails indicate SD.

**Figure 3:**
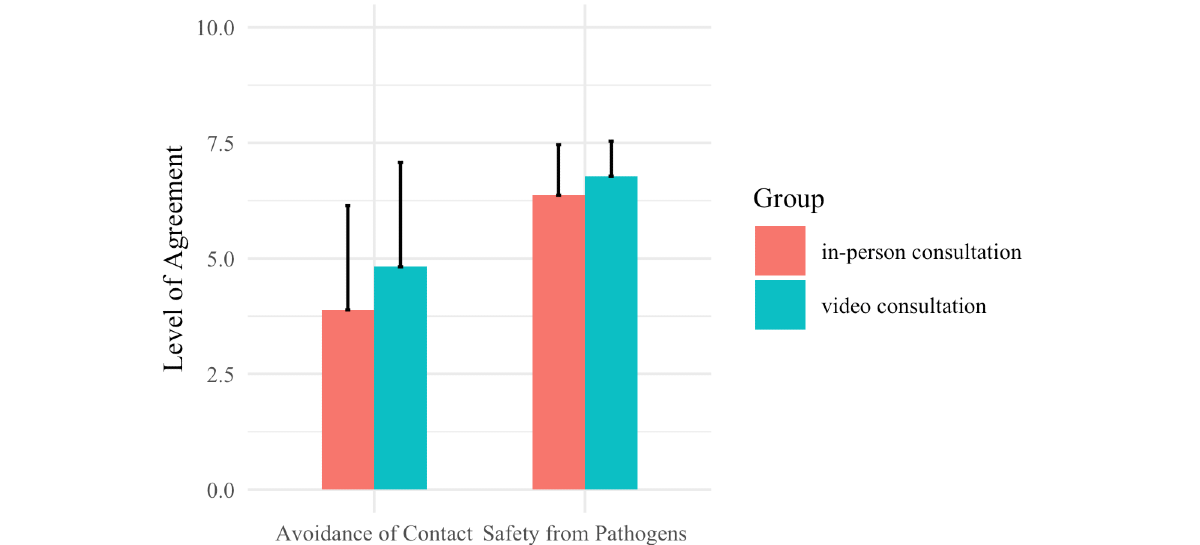
Level of agreement with statements on avoidance of contact with medical professionals during the pandemic (*P*=.045 between groups) and belief in safety from pathogens at home (*P*=.02 between groups). Higher numbers indicate stronger agreement. All ratings were made after either an in-person consultation or a video consultation. The bars indicate mean values and tails indicate SD.

Significant differences between the groups were found in parental age (Figure 1), estimates of waiting time before the appointment (Figure 2), belief in safety from pathogens, and avoidance of contact with medical professionals (Figure 3).

Parents who attended a video consultation were significantly older on average (mean age 33.45, SD 5.11 years) than parents who attended an in-person appointment (mean age 31.4, SD 5.86 years; Shapiro-Wilk *W*=1043; *P*=.044; *d*=–0.1767638). The video-consultation group made estimates of the potential waiting time at the clinic that were significantly longer (mean 23.58, SD 27.75 minutes) than estimates of the potential waiting time at the clinic made by the in-person group (mean 11.39, SD 17.6 minutes; Shapiro-Wilk *W*=1387.5; *P*<.001; *d*=–0.3633795). Parents reported feeling safer from pathogens at home (mean score 6.78, SD 0.76) after a video consultation than after an in-person appointment (mean score 6.37, SD 1.09; Shapiro-Wilk W=1060.5; *P*=.02; d=–0.2577713).

Parents were on average very satisfied with the video and audio quality of the video consultations (mean score 6.29, SD 1.26); they considered examining their children themselves under supervision was on average beneficial (mean score 6.53, SD 1.39) and allowed them to better understand their development (mean score 6.57; SD 1.36); they considered that the video consultations were implemented similarly to in-person appointments (mean score 5.67, SD 1.84); and they accepted video consultations as an alternative in the future and wished to use them again (mean score 6.49; SD 1.09).

Between March 2019 and February 2020, 782 of 984 (79.5%) children born at Essen University Hospital attended a follow-up appointment. During the survey period between March 2020 and February 2021, a total of 788 of 1086 children (73%) attended a follow-up appointment, of which 117 (14.9%) were video consultations.

## Discussion

### Principal Results

The subjective satisfaction of families attending video consultations for follow-up care for their very preterm or at-risk infants was as high as in-person consultations. Parents found the video consultations to be as confidential as in-person appointments. In addition, parents felt comfortable and self-confident performing the guided examinations of their children by themselves during video consultations. Parents indicated that performing the examinations of their children gave them a better understanding of their development. During video consultations, the parents rated the children as being as calm and cooperative as at in-person appointments. Parents rated the feasibility of the video consultations as very high and similar to in-person appointments. Acceptance of the video consultations was considered high, as the parents agreed to choose video consultations again.

During the COVID-19 pandemic, cancellation of in-person appointments due to symptoms of infection with SARS-CoV-2, other viral infections, or quarantine of family members was frequent. However, missed appointments often could not be made up in a timely manner. Dayal et al [12] compared an in-person visit cohort with a telemedicine visit cohort in their outpatient pediatric neurology clinic and reported that telemedicine visits were more likely to be completed versus cancelled or missed compared to in-person visits. The authors concluded that telemedicine could serve as an equal supplement to in-person visits.

### Feasibility of Video Consultations

Parents strongly considered video consultation as an equal alternative for keeping scheduled follow-up appointments. The acceptance of the video consultations by parents and their positive evaluations of the confidentiality of the conversations suggests the possibility of using this modality beyond the pandemic.

Offering video consultations from the beginning of the COVID-19 pandemic was a rapid, adaptive response to meet the needs of the patient population. According to Chew et al [13] the successful deployment of digital tools is dependent on patient willingness to use the tools, provider acceptance, and quality of hardware infrastructure [14].

It is much more difficult for highly complex patients, such as those receiving home oxygen therapy or those who live far away from the hospital, to attend in-person appointments. However, keeping track of patients with ongoing medical and therapeutic requirements reduces the rate of hospitalization. This also holds true for neonatal patients. Robinson et al [15] showed that telemedicine for follow-up care of infants after discharge from the NICU reduced emergency visits to the hospital. Furthermore, it has been reported that 26% of parents receiving telemedicine felt they had more scheduled appointments than necessary, which increased their level of satisfaction [16]. Gund et al [17] showed that telemedicine could reduce the need for home visits among parents practicing neonatal home care. There are examples of randomized controlled trials with promising outcomes for digital tools with ecological validity. The SAVED (Safety and Efficacy of Follow-up for Patients With Abdominal Pain Using Video Consultation) study [18], a randomized controlled trial, showed that video consultation was a safe and efficient tool for follow-up care in patients presenting with acute abdominal pain in the emergency room. Richards et al [19] showed that digital intervention for patients with depression and anxiety was therapeutic and cost-effective and could be a strong tool to counter or account for the treatment gap in mental health. With time- and cost-effective digital tools, diagnosis and treatment may be offered significantly earlier to patients in distress.

Neurodevelopmental follow-up in preterm infants aims to identify children at risk early, in order to initiate intervention to best promote development. De Kleine et al [7] investigated long-term follow-up care of preterm children up to the age of 5 years and revealed a large group of children with developmental disturbances who were previously considered developmentally appropriate. Video consultations offered an opportunity in this study to evaluate development of the children and consult parents about all developmental domains of their child. Gavazzi et al [16] showed the feasibility of video consultations for standardized measurement instruments, such as the Gross Motor Function Measure–88, in a pediatric group with chronical neurological disease. Li et al [20] showed that a combination of follow-up visits that were face-to-face and via video allowed a follow-up rate that was as high during the first wave of the COVID-19 pandemic in April 2020 as in the previous year. In this study, due to the additional offer of a video consultation, the rate of follow-up appointments decreased slightly, but the total number of consultations increased.

During the first 3 infectious peaks of the COVID-19 pandemic in Germany (March 2020-January 2021), there were several weeks when planned in-person appointments for medical causes that were not urgent were prohibited by social distancing rules. This study showed that parents who preferred follow-up care via video consultation were significantly more afraid of an infection and were significantly more likely to avoid contact with medical professionals. Parents of very preterm children have been exceptionally worried about being infected due to the fact that preterm children suffer from respiratory infections more often and have an increased risk of rehospitalization, especially during the first 2 years of life [1]. Brasseler (personal communication, July 8, 2022) showed that parents with preterm infants had more anxiety during the COVID-19 pandemic than parents of infants born at term. Furthermore, participants in the video-consultation group in this study were significantly older than those in the in-person appointment group. A great deal of research has shown that higher maternal age, among other factors, is correlated with higher compliance to follow-up care among very-low-birth weight infants and infants discharged from the NICU [21,22].

In the outpatient clinic, 3 follow-up visits are scheduled in the first year of life and annually until school age (6 years in Germany). The video consultations for follow-up care in this study included patient-reported medical history and a guided examination of the patients by the parents. Observing children in their home environment following parental instructions provided an opportunity to gain insight into the family environment. Standardization was a challenge, and only individual items could be measured. Muscle tone and muscle strength were difficult to examine via video consultation. Nevertheless, observation of the infant’s spontaneous motor skills is one of the most important examinations and is easy to perform via video consultation in most cases. In toddlers and children, an overview of developmental domains in fine and gross motor skills, language and speech, cognition, performance and reasoning, sensory skills, and social-emotional behavior was obtained [7]. The subjective impression of the examiners was also that the children were calmer and more relaxed in their home environment.

### Limitations

This study used a nonrandomized cohort design as the onset of the pandemic required rapid adaptation for developmental neurological follow-up care due to contact restrictions. Due to the completely anonymous collection of the data, no conclusions can be drawn about the families retrospectively. Nevertheless, additional variables would have been of interest from the current perspective, such as the age of the children or the frequency of participation in video consultations. Furthermore, the interviews were conducted after the appointments or consultations and were thus not anonymously registered.

Parents in the in-person appointment group estimated the potential waiting time as being shorter than parents in the video-consultation group. However, technical problems (eg, not enough mobile data available or insufficient battery) also often led to waiting times before video consultations. The actual waiting time was not recorded in either group. Similarly, parents who were offered video consultations but did not accept the offer were not included or evaluated in this study.

### Future Perspectives

The examiners’ perspectives were not elicited but might have revealed valuable new approaches for further research. There appears to be a lack of evidence from research in digital health, which in turn hinders implementation, and vice versa. Future randomized controlled studies are needed for the evaluation of digital tools in medical services offered to chronically ill children [23]. Future studies should also address technological considerations, such as the devices used, data volume, and signal strength.

In developing countries with low incomes and high rural populations, telemedicine could offer, on one hand, opportunities to bring a higher level of health service to patients in isolated regions [12,15], but on the other hand, they might be limited by the supply of hardware and software, as well as by data volume and signal strength; these are issues that should be investigated.

Independent of the pandemic, video consultation has been developed as a low-threshold service to bring medical care closer to the patient. It serves patients very well who live far away from specialists or who have greater logistical challenges, such as children on ventilators [24]. Telemedicine significantly reduces health service use and may therefore reduce health service costs [15,25,26]. Parents feel secure and adequately supported in their own home with their preterm infants and children. This generation of parents has an innate understanding of digital devices and services [27,28]. They feel well informed about the neurodevelopment of their children at risk of neurodevelopmental difficulties. Being closely involved in the examination of their infants during video consultations as part of follow-up care gives parents a better understanding of the neurodevelopment of their children and empowers them as caregivers. Offering follow-up care as a multi-professional team with a combination of in-person visits and interactive media may have the potential to improve the compliance of families and lengthen the period of follow-up care [28]. Overall, telemedicine and video consultations may be used as supplementary tools in medical and follow-up care in the twenty-first century for chronically ill patients and children with the need for continuous attendance and surveillance. The opportunities certainly outweigh the challenges in times such as the COVID-19 pandemic.

### Conclusion

The feasibility of video consultations for follow-up care of very preterm or at-risk infants and parental satisfaction with the consultations was as high as for in-person consultations. Parents rated video consultations as being as confidential as in-person appointments. Telemedicine can be offered as an equivalent alternative to an in-person consultation and is particularly useful under certain circumstances, such as care for very sick children who require assistive devices, such as respiratory support and oxygen, or for children living a long distance away.

## Appendix

Multimedia Appendix 1 Interviews to evaluate the video or in-person consultation of follow-up care of premature infants.

Multimedia Appendix 2 Video consultation in preterm follow-up care during the COVID-19 pandemic -opportunities and challenges.

## Abbreviations

NICU: neonatal intensive care unit

## Data Availability

All data presented in this article and the code for reproducing the statistical results and figures are available in Multimedia Appendix 2.
