# Supplementary material for: Feasibility of Video Consultation for Preterm Neurodevelopmental Follow-up Care During the COVID-19 Pandemic: Cohort Study
Source: JMIR Pediatr Parent. 2023 Jan 25;6:e40940. doi: 10.2196/40940 (PMC9879316; doi:10.2196/40940)
Supplement: Multimedia Appendix 1 [file pediatrics_v6i1e40940_app1.docx]

Appendix 1:

**Interview to evaluate the in person consultation of follow-up care of premature infants.**

1. Relationship to child (e.g., mother/father):
2. Gender:
3. Age:
4. Schooling in years:

1. Have you ever been personally to the Social Pediatric Center (SPC) of the University Medical Center Essen?

a. Yes

b. No

2. How much time did it take you to get to the SPC?

3. What is the distance to the SPC in km?

4. Would you have had to take time off from work for the follow-up appointment?

a. Yes

b. No

c. Other description:

5. How long (minutes) do you think you have had to wait in the SPC's waiting room?

For the next questions, please indicate the number that best describes the aspect of medical care for you. 1 indicates that you strongly disagree with the statement and 7 indicates that you strongly agree with the statement.

6. Taking an active role in my child's health care is important to me.

Strongly disagree ① ② ③ ④ ⑤ ⑥ ⑦ Strongly agree.

7. I was satisfied with the quality of the follow-up appointment.

Strongly disagree ① ② ③ ④ ⑤ ⑥ ⑦ Strongly agree

8. My consultation appointment started on time today.

Don't agree at all ① ② ③ ④ ⑤ ⑥ ⑦ Agree totally

9. My consultation appointment was efficient.

Don't agree at all ① ② ③ ④ ⑤ ⑥ ⑦ Agree completely

10. I believe that the medical examination was conducted in a confidential manner.

Strongly disagree ① ② ③ ④ ⑤ ⑥ ⑦ Strongly agree

11. I was able to share sensitive and/or personal information with my doctor(s)

Strongly disagree ① ② ③ ④ ⑤ ⑥ ⑦ Strongly agree

12. I think the doctor was able to do his/her job.

Strongly disagree ① ② ③ ④ ⑤ ⑥ ⑦ Strongly agree

13. My child was relaxed and cooperative during the examination.

Strongly disagree ① ② ③ ④ ⑤ ⑥ ⑦ Strongly agree

14. The time frame of the consultation appointment was just right.

Do not agree at all ① ② ③ ④ ⑤ ⑥ ⑦ Agree completely

15. The atmosphere during the consultation appointment was very pleasant for me and my child.

Don't agree at all ① ② ③ ④ ⑤ ⑥ ⑦ Agree completely

16. I felt well taken care of by the consultation overall.

Strongly disagree ① ② ③ ④ ⑤ ⑥ ⑦ Strongly agree

17. I was very satisfied with my consultation appointment today.

Don't agree at all ① ② ③ ④ ⑤ ⑥ ⑦ Agree completely

18. I feel safe from potential pathogens at the SPC.

Strongly disagree ① ② ③ ④ ⑤ ⑥ ⑦ Strongly agree

19. I am currently avoiding contact with other people, including medical personnel, during the Corona pandemic.

Don't agree at all ① ② ③ ④ ⑤ ⑥ ⑦ Agree completely

20.I worry about possible infection with Corona virus for myself and my child.

Do not agree at all ① ② ③ ④ ⑤ ⑥ ⑦ Agree completely

**Questionnaire for the evaluation of the video consultation for the follow-up care of premature infants**

1. Relationship to child (e.g., mother/father):
2. Gender:
3. Age:
4. Schooling in years:

1. Have you ever been in person at the Social Pediatric Center (SPC) of the University Medical Center Essen?

a. Yes

b. No

2. How much time would it have taken you to get to the SPC?

3. What is the distance to the SPC in km?

4. Would you have had to take time off from work for the follow-up appointment?

a. Yes

b. No

c. Other description:

5. How long (minutes) do you think you would have had to wait in the SPC's waiting room?

6. Which device did you use to participate in the video consultation?

For the next questions, please circle the number that best describes the aspect of medical care for you. 1 indicates that you strongly disagree with the statement and a 7 indicates that you strongly agree with the statement.

7. Taking an active role in my child's health care is important to me.

Strongly disagree ① ② ③ ④ ⑤ ⑥ ⑦ Strongly agree

8. I was satisfied with the quality of the follow-up appointment.

Strongly disagree ① ② ③ ④ ⑤ ⑥ ⑦ Strongly agree

9. I was satisfied with the video and audio quality.

Strongly disagree ① ② ③ ④ ⑤ ⑥ ⑦ Strongly agree

10. My consultation appointment started on time today.

Strongly disagree ① ② ③ ④ ⑤ ⑥ ⑦ Strongly agree

11. My consultation appointment was efficient.

Strongly disagree ① ② ③ ④ ⑤ ⑥ ⑦ Strongly agree

12. I believe the medical examination was conducted in a confidential manner.

Strongly disagree ① ② ③ ④ ⑤ ⑥ ⑦ Strongly agree

13. I was able to share sensitive and/or personal information with my doctor via video consultation

Strongly disagree ① ② ③ ④ ⑤ ⑥ ⑦ Strongly agree

14. I believe the doctor was able to do his/her job even though a physical examination could not be done.

Strongly disagree ① ② ③ ④ ⑤ ⑥ ⑦ Strongly agree

15. My child was relaxed and cooperative during the video consultation.

Strongly disagree ① ② ③ ④ ⑤ ⑥ ⑦ Strongly agree

16. I felt that "examining" my child myself was very comfortable for my child.

Strongly disagree ① ② ③ ④ ⑤ ⑥ ⑦ Strongly agree

17. The instructions of the position changes and the explanations helped me to better understand the development of my child.

Strongly disagree ① ② ③ ④ ⑤ ⑥ ⑦ Strongly agree

18. The time frame of the video session was just right.

Strongly disagree ① ② ③ ④ ⑤ ⑥ ⑦ Strongly agree

19. The atmosphere during the video consultation was very pleasant for me and my child.

Strongly disagree ① ② ③ ④ ⑤ ⑥ ⑦ Strongly agree

20. I felt well taken care of by the video consultation.

Strongly disagree ① ② ③ ④ ⑤ ⑥ ⑦ Strongly agree

21. I believe that the in-person appointment at the SPC of the University Medical Center Essen does not differ from the video consultation.

Strongly disagree ① ② ③ ④ ⑤ ⑥ ⑦ Strongly agree

22. I was very satisfied with my video consultation today.

Strongly disagree ① ② ③ ④ ⑤ ⑥ ⑦ Strongly agree

23. All in all, I would like to use video consultations in the future.

Strongly disagree ① ② ③ ④ ⑤ ⑥ ⑦ Strongly agree

24. I feel more safe from potential pathogens at the home.

Strongly disagree ① ② ③ ④ ⑤ ⑥ ⑦ Strongly agree

25. I am currently avoiding contact with other people, including medical personnel, during the Corona pandemic.

Don't agree at all ① ② ③ ④ ⑤ ⑥ ⑦ Agree completely

26.I worry about possible infection with Corona virus for myself and my child.

Do not agree at all ① ② ③ ④ ⑤ ⑥ ⑦ Agree completely
