# Supplementary material for: Feasibility of Video Consultation for Preterm Neurodevelopmental Follow-up Care During the COVID-19 Pandemic: Cohort Study
Source: JMIR Pediatr Parent. 2023 Jan 25;6:e40940. doi: 10.2196/40940 (PMC9879316; doi:10.2196/40940)
Supplement: Multimedia Appendix 2 [file pediatrics_v6i1e40940_app2.pdf]

Multimedia Appendix - Video consultation in preterm follow-up care during the COVID-19 pandemic - opportunities and challenges; this is a Multimedia Appendix to a full manuscript published in the J Med Internet Res. For full copyright and citation information see http://dx.doi.org/10.2196/jmir.xxxx (http://dx.doi.org/10.2196/jmir.xxxx)

L. Cordier

July 2022

- 1 DESCRIPTIVE STATISTIC
- 2 normal distribution
  - 2.1 video consulting
  - 2.2 in person appointment
- 3 GROUP DIFFERENCES: Calculated group differences between a video consultation and an outpatient appointment.
  - 3.1 Mann-Whitney U test
  - 3.2 First significant difference
  - 3.3 Second significant difference
  - 3.4 Third significant difference
  - 3.5 Fourth significant difference
- 4 LOCKDOWN
  - 4.1 Distribution over time of interviews conducted.
  - 4.2 Calculation of the differences between the lockdowns
- 5 EXPLORATORY DATA ANALYSIS
  - 5.1 Spearman rank correlations
- 6 Figure

1 DESCRIPTIVE STATISTIC

```
summary(ENN)
```

```
##        ID          Gruppe        ENN_00_Datum_RAW  ENN_00_Datum
##  ENNT001: 1   Min.   :0.0000           :11       Min.   :200107
##  ENNT002: 1   1st Qu.:0.0000   30.04.20: 5       1st Qu.:200509
##  ENNT003: 1   Median :0.0000   25.05.20: 4       Median :200719
##  ENNT004: 1   Mean   :0.3763   08.05.20: 3       Mean   :200846
##  ENNT005: 1   3rd Qu.:1.0000   05.04.20: 2       3rd Qu.:201014
##  ENNT006: 1   Max.   :1.0000   06.10.20: 2       Max.   :210125
##  (Other):87                    (Other) :66       NA's   :11
##  ENN_1a_Gesprächsteilnehmer ENN_1b_Geschlecht  ENN_1c_Alter
##  Min.   :1.000              Min.   :1.000     Min.   :19.00
##  1st Qu.:1.000              1st Qu.:1.000     1st Qu.:30.00
##  Median :1.000              Median :1.000     Median :34.00
##  Mean   :1.033              Mean   :1.033     Mean   :32.73
##  3rd Qu.:1.000              3rd Qu.:1.000     3rd Qu.:36.00
##  Max.   :2.000              Max.   :2.000     Max.   :47.00
##  NA's   :1                  NA's   :1         NA's   :8
##  ENN_1d_Schulbildung_Mutter_in_Jahre ENN_1e_beide_Eltern ENN_2_Endgerät
##  Min.   : 4.00                       Min.   :0.0000      Min.   :1.000
##  1st Qu.:10.00                       1st Qu.:0.0000      1st Qu.:1.000
##  Median :13.00                       Median :1.0000      Median :1.000
##  Mean   :12.68                       Mean   :0.5054      Mean   :1.618
##  3rd Qu.:14.00                       3rd Qu.:1.0000      3rd Qu.:3.000
##  Max.   :19.00                       Max.   :1.0000      Max.   :3.000
##  NA's   :6                                               NA's   :38
##  ENN_3_Bisheriger_persönlicher_Besuch_im_SPZ ENN_4_Zeit_für_Anfahrt
##  Min.   : 0.0000                             Min.   : 4.00
##  1st Qu.: 0.0000                             1st Qu.:20.00
##  Median : 1.0000                             Median :30.00
##  Mean   : 0.7742                             Mean   :28.79
##  3rd Qu.: 1.0000                             3rd Qu.:37.50
##  Max.   :10.0000                             Max.   :90.00
##                                              NA's   :2
##  ENN_5_Entfernung_km ENN_6_Urlaub_nehmen_für_Termin ENN_7_Potentielle_Wartezeit
##  Min.   : 1.00       Min.   : 0.000                 Min.   :  0.00
##  1st Qu.: 5.00       1st Qu.: 0.000                 1st Qu.:  5.00
##  Median :10.00       Median : 0.000                 Median : 10.00
##  Mean   :15.86       Mean   : 1.283                 Mean   : 19.11
##  3rd Qu.:21.25       3rd Qu.: 1.000                 3rd Qu.: 30.00
##  Max.   :75.00       Max.   :60.000                 Max.   :180.00
##  NA's   :5           NA's   :1                      NA's   :3
##  ENN_8_Aktive_Rolle_Gesundheit_Kind ENN_9_Qualität_ENN
##  Min.   : 1.000                     Min.   :5.000
##  1st Qu.: 7.000                     1st Qu.:7.000
##  Median : 7.000                     Median :7.000
##  Mean   : 7.226                     Mean   :6.882
##  3rd Qu.: 7.000                     3rd Qu.:7.000
##  Max.   :20.000                     Max.   :7.000
##
##  ENN_10_Zufriedenheit_Video_Audio ENN_11_Pünktlichkeit ENN_12_Effizienz
##  Min.   :2.000                    Min.   :1.000        Min.   :5.000
##  1st Qu.:6.000                    1st Qu.:6.000        1st Qu.:7.000
##  Median :7.000                    Median :7.000        Median :7.000
##  Mean   :6.359                    Mean   :6.506        Mean   :6.882
##  3rd Qu.:7.000                    3rd Qu.:7.000        3rd Qu.:7.000
##  Max.   :7.000                    Max.   :7.000        Max.   :7.000
```

```
##  NA's   :29                            NA's   :4
##  ENN_13_Vertrauliche_Weise ENN_14__Sensible_Info ENN_15_Kind_beurteilbar
##  Min.   :6.000             Min.   :6.000         Min.   :4.000
##  1st Qu.:7.000             1st Qu.:7.000         1st Qu.:7.000
##  Median :7.000             Median :7.000         Median :7.000
##  Mean   :6.946             Mean   :6.946         Mean   :6.828
##  3rd Qu.:7.000             3rd Qu.:7.000         3rd Qu.:7.000
##  Max.   :7.000             Max.   :7.000         Max.   :7.000
##
##  ENN_16_Entspannung_und_Kooperation ENN_17_selber_untersuchen
##  Min.   :0.000                      Min.   :0.000
##  1st Qu.:6.000                      1st Qu.:7.000
##  Median :7.000                      Median :7.000
##  Mean   :6.409                      Mean   :6.492
##  3rd Qu.:7.000                      3rd Qu.:7.000
##  Max.   :7.000                      Max.   :7.000
##                                     NA's   :32
##  ENN_18_Positionswechsel_Erklärungen ENN_19_Zeitlicher_Umfang ENN_20_Atmosphäre
##  Min.   :0.000                       Min.   :3.000            Min.   :1.000
##  1st Qu.:7.000                       1st Qu.:7.000            1st Qu.:7.000
##  Median :7.000                       Median :7.000            Median :7.000
##  Mean   :6.571                       Mean   :6.807            Mean   :6.828
##  3rd Qu.:7.000                       3rd Qu.:7.000            3rd Qu.:7.000
##  Max.   :7.000                       Max.   :7.000            Max.   :7.000
##  NA's   :37                          NA's   :5
##  ENN_21_gute_Betreuung ENN_27_Zufriedenheit_Insgesamt
##  Min.   :6.000         Min.   :5.000
##  1st Qu.:7.000         1st Qu.:7.000
##  Median :7.000         Median :7.000
##  Mean   :6.946         Mean   :6.924
##  3rd Qu.:7.000         3rd Qu.:7.000
##  Max.   :7.000         Max.   :7.000
##                        NA's   :1
##  ENN_22_Häusliche_Umgebung_Sicherer_vor_Erregern
##  Min.   :3.000
##  1st Qu.:7.000
##  Median :7.000
##  Mean   :6.612
##  3rd Qu.:7.000
##  Max.   :7.000
##  NA's   :8
##  ENN_23_Kontaktvermeidung_andere_Menschen
##  Min.   :1.000
##  1st Qu.:5.000
##  Median :6.000
##  Mean   :5.643
##  3rd Qu.:7.000
##  Max.   :7.000
##  NA's   :9
##  ENN_24_Kontaktvermeidung_medizinisches_Personal ENN_25_Sorge_vor_Corona
##  Min.   :1.000                                   Min.   :1.000
##  1st Qu.:3.000                                   1st Qu.:5.000
##  Median :5.000                                   Median :7.000
##  Mean   :4.435                                   Mean   :5.635
##  3rd Qu.:7.000                                   3rd Qu.:7.000
##  Max.   :7.000                                   Max.   :7.000
```

```
##  NA's   :8                                       NA's   :8
##  ENN_26_Videosprechstunde_ähnlich_Termin_vor_Ort ENN_28_Zukünftiger_Anspruch
##  Min.   :1.000                                   Min.   :2.000
##  1st Qu.:5.000                                   1st Qu.:7.000
##  Median :7.000                                   Median :7.000
##  Mean   :5.672                                   Mean   :6.491
##  3rd Qu.:7.000                                   3rd Qu.:7.000
##  Max.   :7.000                                   Max.   :7.000
##  NA's   :32                                      NA's   :36
##        X
##  Min.   :7
##  1st Qu.:7
##  Median :7
##  Mean   :7
##  3rd Qu.:7
##  Max.   :7
##  NA's   :91
```

```
describe(ENN)
```

```
## ENN
##
##  37  Variables      93  Observations
## ---------------------------------------------------------------------------------
## ID
##        n  missing distinct
##       93        0       93
##
## lowest : ENNT001 ENNT002 ENNT003 ENNT004 ENNT005
## highest: ENNV054 ENNV055 ENNV056 ENNV057 ENNV058
## ---------------------------------------------------------------------------------
## Gruppe
##        n  missing distinct     Info      Sum     Mean      Gmd
##       93        0        2    0.704       35   0.3763   0.4745
##
## ---------------------------------------------------------------------------------
## ENN_00_Datum_RAW
##        n  missing distinct
##       93        0       57
##
## lowest :          05.04.20 05.05.20 05.11.20 06.10.20
## highest: 29.06.20 30.04.20 30.07.20 30.10.20 30.11.20
## ---------------------------------------------------------------------------------
## ENN_00_Datum
##        n  missing distinct     Info     Mean      Gmd      .05      .10
##       82       11       56    0.999   200846    556.2   200307   200408
##      .25      .50      .75      .90      .95
##   200509   200719   201014   201120   201126
##
## lowest : 200107 200207 200307 200405 200408, highest: 201126 201130 201214 201216
210125
## ---------------------------------------------------------------------------------
## ENN_1a_Gesprächsteilnehmer
##        n  missing distinct     Info     Mean      Gmd
##       92        1        2    0.095    1.033  0.06378
##
## Value          1     2
## Frequency     89     3
## Proportion 0.967 0.033
## ---------------------------------------------------------------------------------
## ENN_1b_Geschlecht
##        n  missing distinct     Info     Mean      Gmd
##       92        1        2    0.095    1.033  0.06378
##
## Value          1     2
## Frequency     89     3
## Proportion 0.967 0.033
## ---------------------------------------------------------------------------------
## ENN_1c_Alter
##        n  missing distinct     Info     Mean      Gmd      .05      .10
##       85        8       24    0.992    32.73     6.08     22.0     25.4
##      .25      .50      .75      .90      .95
##     30.0     34.0     36.0     39.0     40.0
##
## lowest : 19 20 21 22 23, highest: 39 40 41 42 47
```

```
## --------------------------------------------------------------------------------
## ENN_1d_Schulbildung_Mutter_in_Jahre
##        n  missing distinct     Info     Mean      Gmd      .05      .10
##       87        6       13    0.954    12.68    3.107      9.3     10.0
##      .25      .50      .75      .90      .95
##     10.0     13.0     14.0     16.4     18.0
##
## lowest :  4  8  9 10 11, highest: 15 16 17 18 19
##
## Value          4     8     9    10    11    12    13    14    15    16    17
## Frequency      2     2     1    19     2    10    28     3     6     5     2
## Proportion 0.023 0.023 0.011 0.218 0.023 0.115 0.322 0.034 0.069 0.057 0.023
##
## Value         18    19
## Frequency      5     2
## Proportion 0.057 0.023
## --------------------------------------------------------------------------------
## ENN_1e_beide_Eltern
##        n  missing distinct     Info      Sum     Mean      Gmd
##       93        0        2     0.75       47   0.5054   0.5054
##
## --------------------------------------------------------------------------------
## ENN_2_Endgerät
##        n  missing distinct     Info     Mean      Gmd
##       55       38        3    0.699    1.618   0.8646
##
## Value          1     2     3
## Frequency     36     4    15
## Proportion 0.655 0.073 0.273
## --------------------------------------------------------------------------------
## ENN_3_Bisheriger_persönlicher_Besuch_im_SPZ
##        n  missing distinct     Info     Mean      Gmd
##       93        0        3     0.67   0.7742   0.6353
##
## Value          0     1    10
## Frequency     30    62     1
## Proportion 0.323 0.667 0.011
## --------------------------------------------------------------------------------
## ENN_4_Zeit_für_Anfahrt
##        n  missing distinct     Info     Mean      Gmd      .05      .10
##       91        2       16    0.971    28.79    17.07     10.0     10.0
##      .25      .50      .75      .90      .95
##     20.0     30.0     37.5     45.0     60.0
##
## lowest :  4  5 10 12 14, highest: 45 50 60 75 90
##
## Value          4     5    10    12    14    15    20    25    30    35    40
## Frequency      1     3     9     1     1     4    18     6    24     1     9
## Proportion 0.011 0.033 0.099 0.011 0.011 0.044 0.198 0.066 0.264 0.011 0.099
##
## Value         45    50    60    75    90
## Frequency      5     1     6     1     1
## Proportion 0.055 0.011 0.066 0.011 0.011
## --------------------------------------------------------------------------------
## ENN_5_Entfernung_km
##        n  missing distinct     Info     Mean      Gmd      .05      .10
```

```
##       88        5       31    0.995    15.86    15.42     2.00     2.00 
##      .25      .50      .75      .90      .95 
##     5.00    10.00    21.25    35.90    40.00 
## 
## lowest :  1  2  3  4  5, highest: 40 45 60 70 75
## --------------------------------------------------------------------------------
## ENN_6_Urlaub_nehmen_für_Termin 
##        n  missing distinct     Info     Mean      Gmd 
##       92        1        6    0.749    1.283    2.224 
## 
## lowest :  0  1  2  4  5, highest:  1  2  4  5 60
##                                               
## Value          0     1     2     4     5    60
## Frequency     56    27     2     3     3     1
## Proportion 0.609 0.293 0.022 0.033 0.033 0.011
## --------------------------------------------------------------------------------
## ENN_7_Potentielle_Wartezeit 
##        n  missing distinct     Info     Mean      Gmd      .05      .10 
##       90        3       14    0.978    19.11    21.88        0        0 
##      .25      .50      .75      .90      .95 
##        5       10       30       45       60 
## 
## lowest :   0   1   5   7  10, highest:  45  60  70  90 180
##                                                                             
## Value          0     1     5     7    10    15    20    30    40    45    60
## Frequency     12     4    14     3    19     7     4    16     1     2     5
## Proportion 0.133 0.044 0.156 0.033 0.211 0.078 0.044 0.178 0.011 0.022 0.056
##                         
## Value         70    90   180
## Frequency      1     1     1
## Proportion 0.011 0.011 0.011
## --------------------------------------------------------------------------------
## ENN_8_Aktive_Rolle_Gesundheit_Kind 
##        n  missing distinct     Info     Mean      Gmd 
##       93        0        6    0.263    7.226   0.9065 
## 
## lowest :  1  5  6  7 10, highest:  5  6  7 10 20
##                                               
## Value          1     5     6     7    10    20
## Frequency      1     1     3    84     2     2
## Proportion 0.011 0.011 0.032 0.903 0.022 0.022
## --------------------------------------------------------------------------------
## ENN_9_Qualität_ENN 
##        n  missing distinct     Info     Mean      Gmd 
##       93        0        3    0.236    6.882   0.2221 
##                             
## Value          5     6     7
## Frequency      3     5    85
## Proportion 0.032 0.054 0.914
## --------------------------------------------------------------------------------
## ENN_10_Zufriedenheit_Video_Audio 
##        n  missing distinct     Info     Mean      Gmd 
##       64       29        6    0.602    6.359    1.038 
## 
## lowest : 2 3 4 5 6, highest: 3 4 5 6 7
## 
```

```
## Value          2     3     4     5     6     7
## Frequency      1     2     3     8     3    47
## Proportion 0.016 0.031 0.047 0.125 0.047 0.734
## ---------------------------------------------------------------------------------
## ENN_11_Pünktlichkeit
##        n  missing distinct     Info     Mean      Gmd
##       89        4        6    0.623    6.506   0.8069
##
## lowest : 1 3 4 5 6, highest: 3 4 5 6 7
##
## Value          1     3     4     5     6     7
## Frequency      1     1     3     5    15    64
## Proportion 0.011 0.011 0.034 0.056 0.169 0.719
## ---------------------------------------------------------------------------------
## ENN_12_Effizienz
##        n  missing distinct     Info     Mean      Gmd
##       93        0        3    0.288    6.882   0.2155
##
## Value          5     6     7
## Frequency      1     9    83
## Proportion 0.011 0.097 0.892
## ---------------------------------------------------------------------------------
## ENN_13_Vertrauliche_Weise
##        n  missing distinct     Info     Mean      Gmd
##       93        0        2    0.153    6.946   0.1029
##
## Value          6     7
## Frequency      5    88
## Proportion 0.054 0.946
## ---------------------------------------------------------------------------------
## ENN_14__Sensible_Info
##        n  missing distinct     Info     Mean      Gmd
##       93        0        2    0.153    6.946   0.1029
##
## Value          6     7
## Frequency      5    88
## Proportion 0.054 0.946
## ---------------------------------------------------------------------------------
## ENN_15_Kind_beurteilbar
##        n  missing distinct     Info     Mean      Gmd
##       93        0        4    0.263    6.828   0.3221
##
## Value          4     5     6     7
## Frequency      2     3     4    84
## Proportion 0.022 0.032 0.043 0.903
## ---------------------------------------------------------------------------------
## ENN_16_Entspannung_und_Kooperation
##        n  missing distinct     Info     Mean      Gmd
##       93        0        6    0.683    6.409   0.9327
##
## lowest : 0 3 4 5 6, highest: 3 4 5 6 7
##
## Value          0     3     4     5     6     7
## Frequency      1     1     5     6    17    63
## Proportion 0.011 0.011 0.054 0.065 0.183 0.677
## ---------------------------------------------------------------------------------
```

```
## ENN_17_selber_untersuchen
##        n  missing distinct     Info     Mean      Gmd
##       61       32        5     0.54    6.492   0.8929
##
## lowest : 0 4 5 6 7, highest: 0 4 5 6 7
##
## Value          0     4     5     6     7
## Frequency      2     1     3     8    47
## Proportion 0.033 0.016 0.049 0.131 0.770
## ---------------------------------------------------------------------------
## ENN_18_Positionswechsel_Erklärungen
##        n  missing distinct     Info     Mean      Gmd
##       56       37        4    0.445    6.571   0.7844
##
## Value          0     5     6     7
## Frequency      2     2     6    46
## Proportion 0.036 0.036 0.107 0.821
## ---------------------------------------------------------------------------
## ENN_19_Zeitlicher_Umfang
##        n  missing distinct     Info     Mean      Gmd
##       88        5        4    0.355    6.807   0.3503
##
## Value          3     5     6     7
## Frequency      1     2     9    76
## Proportion 0.011 0.023 0.102 0.864
## ---------------------------------------------------------------------------
## ENN_20_Atmosphäre
##        n  missing distinct     Info     Mean      Gmd
##       93        0        4    0.236    6.828   0.3282
##
## Value          1     5     6     7
## Frequency      1     3     4    85
## Proportion 0.011 0.032 0.043 0.914
## ---------------------------------------------------------------------------
## ENN_21_gute_Betreuung
##        n  missing distinct     Info     Mean      Gmd
##       93        0        2    0.153    6.946   0.1029
##
## Value          6     7
## Frequency      5    88
## Proportion 0.054 0.946
## ---------------------------------------------------------------------------
## ENN_27_Zufriedenheit_Insgesamt
##        n  missing distinct     Info     Mean      Gmd
##       92        1        3    0.183    6.924    0.145
##
## Value          5     6     7
## Frequency      1     5    86
## Proportion 0.011 0.054 0.935
## ---------------------------------------------------------------------------
## ENN_22_Häusliche_Umgebung_Sicherer_vor_Erregern
##        n  missing distinct     Info     Mean      Gmd
##       85        8        5    0.465    6.612   0.6779
##
## lowest : 3 4 5 6 7, highest: 3 4 5 6 7
##
```

```
## Value          3     4     5     6     7
## Frequency      2     3     5     6    69
## Proportion 0.024 0.035 0.059 0.071 0.812
## ---------------------------------------------------------------------------------
## ENN_23_Kontaktvermeidung_andere_Menschen
##        n  missing distinct     Info     Mean      Gmd
##       84        9        7    0.918    5.643    1.605
##
## lowest : 1 2 3 4 5, highest: 3 4 5 6 7
##
## Value          1     2     3     4     5     6     7
## Frequency      2     2     3    11    15    17    34
## Proportion 0.024 0.024 0.036 0.131 0.179 0.202 0.405
## ---------------------------------------------------------------------------------
## ENN_24_Kontaktvermeidung_medizinisches_Personal
##        n  missing distinct     Info     Mean      Gmd
##       85        8        7    0.954    4.435    2.559
##
## lowest : 1 2 3 4 5, highest: 3 4 5 6 7
##
## Value          1     2     3     4     5     6     7
## Frequency     20     1     6     9    17     8    24
## Proportion 0.235 0.012 0.071 0.106 0.200 0.094 0.282
## ---------------------------------------------------------------------------------
## ENN_25_Sorge_vor_Corona
##        n  missing distinct     Info     Mean      Gmd
##       85        8        7    0.846    5.635    1.862
##
## lowest : 1 2 3 4 5, highest: 3 4 5 6 7
##
## Value          1     2     3     4     5     6     7
## Frequency      6     1     5     7    13     8    45
## Proportion 0.071 0.012 0.059 0.082 0.153 0.094 0.529
## ---------------------------------------------------------------------------------
## ENN_26_Videosprechstunde_ähnlich_Termin_vor_Ort
##        n  missing distinct     Info     Mean      Gmd
##       61       32        7     0.85    5.672     1.82
##
## lowest : 1 2 3 4 5, highest: 3 4 5 6 7
##
## Value          1     2     3     4     5     6     7
## Frequency      4     1     2     7     8     7    32
## Proportion 0.066 0.016 0.033 0.115 0.131 0.115 0.525
## ---------------------------------------------------------------------------------
## ENN_28_Zukünftiger_Anspruch
##        n  missing distinct     Info     Mean      Gmd
##       57       36        6    0.538    6.491   0.8584
##
## lowest : 2 3 4 5 6, highest: 3 4 5 6 7
##
## Value          2     3     4     5     6     7
## Frequency      1     1     1     7     3    44
## Proportion 0.018 0.018 0.018 0.123 0.053 0.772
## ---------------------------------------------------------------------------------
## X
##        n  missing distinct     Info     Mean      Gmd
```

```
##        2       91        1        0        7        0
##
## Value      7
## Frequency  2
## Proportion 1
## ---------------------------------------------------------------------------------
```

```
describeBy(ENN, group="Gruppe")
```

```
## Warning in FUN(newX[, i], ...): kein nicht-fehlendes Argument für min; gebe Inf
## zurück

## Warning in FUN(newX[, i], ...): kein nicht-fehlendes Argument für min; gebe Inf
## zurück

## Warning in FUN(newX[, i], ...): kein nicht-fehlendes Argument für min; gebe Inf
## zurück

## Warning in FUN(newX[, i], ...): kein nicht-fehlendes Argument für min; gebe Inf
## zurück
```

```
## Warning in FUN(newX[, i], ...): kein nicht-fehlendes Argument für max; gebe -Inf
## zurück

## Warning in FUN(newX[, i], ...): kein nicht-fehlendes Argument für max; gebe -Inf
## zurück

## Warning in FUN(newX[, i], ...): kein nicht-fehlendes Argument für max; gebe -Inf
## zurück

## Warning in FUN(newX[, i], ...): kein nicht-fehlendes Argument für max; gebe -Inf
## zurück
```

```
##
##  Descriptive statistics by group
## Gruppe: 0
##                                                vars  n      mean     sd
## ID*                                               1 58     64.50  16.89
## Gruppe                                            2 58      0.00   0.00
## ENN_00_Datum_RAW*                                 3 58     28.19  19.98
## ENN_00_Datum                                      4 50 200766.80 311.96
## ENN_1a_Gesprächsteilnehmer                        5 58      1.03   0.18
## ENN_1b_Geschlecht                                 6 58      1.03   0.18
## ENN_1c_Alter                                      7 55     33.45   5.11
## ENN_1d_Schulbildung_Mutter_in_Jahre               8 57     12.40   2.82
## ENN_1e_beide_Eltern                               9 58      0.60   0.49
## ENN_2_Endgerät                                   10 55      1.62   0.89
## ENN_3_Bisheriger_persönlicher_Besuch_im_SPZ      11 58      0.72   1.33
## ENN_4_Zeit_für_Anfahrt                           12 57     28.58  16.22
## ENN_5_Entfernung_km                              13 54     15.37  14.53
## ENN_6_Urlaub_nehmen_für_Termin                   14 58      1.59   7.87
## ENN_7_Potentielle_Wartezeit                      15 57     23.58  27.75
## ENN_8_Aktive_Rolle_Gesundheit_Kind               16 58      7.40   2.41
## ENN_9_Qualität_ENN                               17 58      6.86   0.44
## ENN_10_Zufriedenheit_Video_Audio                 18 58      6.29   1.26
## ENN_11_Pünktlichkeit                             19 58      6.47   1.14
## ENN_12_Effizienz                                 20 58      6.86   0.40
## ENN_13_Vertrauliche_Weise                        21 58      6.95   0.22
## ENN_14__Sensible_Info                            22 58      6.93   0.26
## ENN_15_Kind_beurteilbar                          23 58      6.76   0.71
## ENN_16_Entspannung_und_Kooperation               24 58      6.45   1.17
## ENN_17_selber_untersuchen                        25 57      6.53   1.39
## ENN_18_Positionswechsel_Erklärungen              26 56      6.57   1.36
## ENN_19_Zeitlicher_Umfang                         27 57      6.86   0.40
## ENN_20_Atmosphäre                                28 58      6.90   0.41
## ENN_21_gute_Betreuung                            29 58      6.95   0.22
## ENN_27_Zufriedenheit_Insgesamt                   30 57      6.89   0.36
## ENN_22_Häusliche_Umgebung_Sicherer_vor_Erregern  31 50      6.78   0.76
## ENN_23_Kontaktvermeidung_andere_Menschen         32 49      5.78   1.40
## ENN_24_Kontaktvermeidung_medizinisches_Personal  33 50      4.82   2.26
## ENN_25_Sorge_vor_Corona                          34 50      5.86   1.69
## ENN_26_Videosprechstunde_ähnlich_Termin_vor_Ort  35 57      5.67   1.84
## ENN_28_Zukünftiger_Anspruch                      36 57      6.49   1.09
## X                                                37  2      7.00   0.00
##                                                  median   trimmed    mad
## ID*                                                64.5     64.50  21.50
## Gruppe                                              0.0      0.00   0.00
## ENN_00_Datum_RAW*                                  32.5     28.19  23.72
## ENN_00_Datum                                   200868.0 200789.45 368.43
## ENN_1a_Gesprächsteilnehmer                          1.0      1.00   0.00
## ENN_1b_Geschlecht                                   1.0      1.00   0.00
## ENN_1c_Alter                                       35.0     33.84   4.45
## ENN_1d_Schulbildung_Mutter_in_Jahre                13.0     12.32   1.48
## ENN_1e_beide_Eltern                                 1.0      0.62   0.00
## ENN_2_Endgerät                                      1.0      1.53   0.00
## ENN_3_Bisheriger_persönlicher_Besuch_im_SPZ         1.0      0.58   0.00
## ENN_4_Zeit_für_Anfahrt                             30.0     27.23  14.83
## ENN_5_Entfernung_km                                10.0     13.32  11.12
```

```
## ENN_6_Urlaub_nehmen_für_Termin                           0.0       0.40   0.00
## ENN_7_Potentielle_Wartezeit                             15.0      18.66  14.83
## ENN_8_Aktive_Rolle_Gesundheit_Kind                       7.0       7.00   0.00
## ENN_9_Qualität_ENN                                       7.0       6.98   0.00
## ENN_10_Zufriedenheit_Video_Audio                         7.0       6.54   0.00
## ENN_11_Pünktlichkeit                                     7.0       6.73   0.00
## ENN_12_Effizienz                                         7.0       6.96   0.00
## ENN_13_Vertrauliche_Weise                                7.0       7.00   0.00
## ENN_14__Sensible_Info                                    7.0       7.00   0.00
## ENN_15_Kind_beurteilbar                                  7.0       6.96   0.00
## ENN_16_Entspannung_und_Kooperation                       7.0       6.69   0.00
## ENN_17_selber_untersuchen                                7.0       6.87   0.00
## ENN_18_Positionswechsel_Erklärungen                      7.0       6.89   0.00
## ENN_19_Zeitlicher_Umfang                                 7.0       6.96   0.00
## ENN_20_Atmosphäre                                        7.0       7.00   0.00
## ENN_21_gute_Betreuung                                    7.0       7.00   0.00
## ENN_27_Zufriedenheit_Insgesamt                           7.0       7.00   0.00
## ENN_22_Häusliche_Umgebung_Sicherer_vor_Erregern          7.0       7.00   0.00
## ENN_23_Kontaktvermeidung_andere_Menschen                 6.0       5.95   1.48
## ENN_24_Kontaktvermeidung_medizinisches_Personal          5.0       5.03   2.97
## ENN_25_Sorge_vor_Corona                                  7.0       6.22   0.00
## ENN_26_Videosprechstunde_ähnlich_Termin_vor_Ort          7.0       6.00   0.00
## ENN_28_Zukünftiger_Anspruch                              7.0       6.72   0.00
## X                                                        7.0       7.00   0.00
##                                                        min    max range  skew
## ID*                                                     36     93    57  0.00
## Gruppe                                                   0      0     0   NaN
## ENN_00_Datum_RAW*                                        1     57    56 -0.11
## ENN_00_Datum                                        200107 201130  1023 -0.39
## ENN_1a_Gesprächsteilnehmer                               1      2     1  4.97
## ENN_1b_Geschlecht                                        1      2     1  4.97
## ENN_1c_Alter                                            21     42    21 -0.68
## ENN_1d_Schulbildung_Mutter_in_Jahre                      4     19    15 -0.20
## ENN_1e_beide_Eltern                                      0      1     1 -0.41
## ENN_2_Endgerät                                           1      3     2  0.80
## ENN_3_Bisheriger_persönlicher_Besuch_im_SPZ              0     10    10  5.72
## ENN_4_Zeit_für_Anfahrt                                   4     90    86  1.07
## ENN_5_Entfernung_km                                      1     70    69  1.40
## ENN_6_Urlaub_nehmen_für_Termin                           0     60    60  7.05
## ENN_7_Potentielle_Wartezeit                              1    180   179  3.45
## ENN_8_Aktive_Rolle_Gesundheit_Kind                       6     20    14  4.90
## ENN_9_Qualität_ENN                                       5      7     2 -3.16
## ENN_10_Zufriedenheit_Video_Audio                         2      7     5 -1.65
## ENN_11_Pünktlichkeit                                     1      7     6 -2.76
## ENN_12_Effizienz                                         5      7     2 -2.84
## ENN_13_Vertrauliche_Weise                                6      7     1 -3.94
## ENN_14__Sensible_Info                                    6      7     1 -3.31
## ENN_15_Kind_beurteilbar                                  4      7     3 -2.83
## ENN_16_Entspannung_und_Kooperation                       0      7     7 -3.28
## ENN_17_selber_untersuchen                                0      7     7 -3.76
## ENN_18_Positionswechsel_Erklärungen                      0      7     7 -4.07
## ENN_19_Zeitlicher_Umfang                                 5      7     2 -2.81
## ENN_20_Atmosphäre                                        5      7     2 -3.88
## ENN_21_gute_Betreuung                                    6      7     1 -3.94
## ENN_27_Zufriedenheit_Insgesamt                           5      7     2 -3.53
## ENN_22_Häusliche_Umgebung_Sicherer_vor_Erregern          3      7     4 -3.66
```

```
## ENN_23_Kontaktvermeidung_andere_Menschen           1      7     6 -1.11
## ENN_24_Kontaktvermeidung_medizinisches_Personal    1      7     6 -0.62
## ENN_25_Sorge_vor_Corona                            1      7     6 -1.55
## ENN_26_Videosprechstunde_ähnlich_Termin_vor_Ort    1      7     6 -1.28
## ENN_28_Zukünftiger_Anspruch                        2      7     5 -2.27
## X                                                  7      7     0   NaN
##                                              kurtosis    se
## ID*                                             -1.26  2.22
## Gruppe                                            NaN  0.00
## ENN_00_Datum_RAW*                               -1.55  2.62
## ENN_00_Datum                                    -1.20 44.12
## ENN_1a_Gesprächsteilnehmer                      23.11  0.02
## ENN_1b_Geschlecht                               23.11  0.02
## ENN_1c_Alter                                    -0.19  0.69
## ENN_1d_Schulbildung_Mutter_in_Jahre              1.67  0.37
## ENN_1e_beide_Eltern                             -1.86  0.06
## ENN_2_Endgerät                                  -1.28  0.12
## ENN_3_Bisheriger_persönlicher_Besuch_im_SPZ     37.22  0.18
## ENN_4_Zeit_für_Anfahrt                           2.04  2.15
## ENN_5_Entfernung_km                              1.95  1.98
## ENN_6_Urlaub_nehmen_für_Termin                  49.40  1.03
## ENN_7_Potentielle_Wartezeit                     15.56  3.68
## ENN_8_Aktive_Rolle_Gesundheit_Kind              22.67  0.32
## ENN_9_Qualität_ENN                               9.39  0.06
## ENN_10_Zufriedenheit_Video_Audio                 1.77  0.17
## ENN_11_Pünktlichkeit                             8.41  0.15
## ENN_12_Effizienz                                 7.87  0.05
## ENN_13_Vertrauliche_Weise                       13.79  0.03
## ENN_14__Sensible_Info                            9.14  0.03
## ENN_15_Kind_beurteilbar                          6.94  0.09
## ENN_16_Entspannung_und_Kooperation              13.67  0.15
## ENN_17_selber_untersuchen                       14.30  0.18
## ENN_18_Positionswechsel_Erklärungen             16.55  0.18
## ENN_19_Zeitlicher_Umfang                         7.65  0.05
## ENN_20_Atmosphäre                               14.30  0.05
## ENN_21_gute_Betreuung                           13.79  0.03
## ENN_27_Zufriedenheit_Insgesamt                  12.66  0.05
## ENN_22_Häusliche_Umgebung_Sicherer_vor_Erregern 13.15  0.11
## ENN_23_Kontaktvermeidung_andere_Menschen         0.94  0.20
## ENN_24_Kontaktvermeidung_medizinisches_Personal -1.11  0.32
## ENN_25_Sorge_vor_Corona                          1.64  0.24
## ENN_26_Videosprechstunde_ähnlich_Termin_vor_Ort  0.59  0.24
## ENN_28_Zukünftiger_Anspruch                      4.92  0.14
## X                                                 NaN  0.00
## ------------------------------------------------------------
## Gruppe: 1
##                                              vars  n      mean      sd
## ID*                                             1 35     18.00   10.25
## Gruppe                                          2 35      1.00    0.00
## ENN_00_Datum_RAW*                               3 35     22.26   15.95
## ENN_00_Datum                                    4 32 200970.41 1688.90
## ENN_1a_Gesprächsteilnehmer                      5 34      1.03    0.17
## ENN_1b_Geschlecht                               6 34      1.03    0.17
## ENN_1c_Alter                                    7 30     31.40    5.86
## ENN_1d_Schulbildung_Mutter_in_Jahre             8 30     13.20    2.94
## ENN_1e_beide_Eltern                             9 35      0.34    0.48
```

```
## ENN_2_Endgerät                                  10  0         NaN       NA
## ENN_3_Bisheriger_persönlicher_Besuch_im_SPZ     11 35        0.86     0.36
## ENN_4_Zeit_für_Anfahrt                          12 34       29.15    15.74
## ENN_5_Entfernung_km                             13 34       16.65    16.34
## ENN_6_Urlaub_nehmen_für_Termin                  14 34        0.76     1.39
## ENN_7_Potentielle_Wartezeit                     15 33       11.39    17.60
## ENN_8_Aktive_Rolle_Gesundheit_Kind              16 35        6.94     1.30
## ENN_9_Qualität_ENN                              17 35        6.91     0.37
## ENN_10_Zufriedenheit_Video_Audio                18  6        7.00     0.00
## ENN_11_Pünktlichkeit                            19 31        6.58     0.76
## ENN_12_Effizienz                                20 35        6.91     0.28
## ENN_13_Vertrauliche_Weise                       21 35        6.94     0.24
## ENN_14__Sensible_Info                           22 35        6.97     0.17
## ENN_15_Kind_beurteilbar                         23 35        6.94     0.24
## ENN_16_Entspannung_und_Kooperation              24 35        6.34     1.08
## ENN_17_selber_untersuchen                       25  4        6.00     0.82
## ENN_18_Positionswechsel_Erklärungen             26  0         NaN       NA
## ENN_19_Zeitlicher_Umfang                        27 31        6.71     0.82
## ENN_20_Atmosphäre                               28 35        6.71     1.07
## ENN_21_gute_Betreuung                           29 35        6.94     0.24
## ENN_27_Zufriedenheit_Insgesamt                  30 35        6.97     0.17
## ENN_22_Häusliche_Umgebung_Sicherer_vor_Erregern 31 35        6.37     1.09
## ENN_23_Kontaktvermeidung_andere_Menschen        32 35        5.46     1.67
## ENN_24_Kontaktvermeidung_medizinisches_Personal 33 35        3.89     2.25
## ENN_25_Sorge_vor_Corona                         34 35        5.31     2.03
## ENN_26_Videosprechstunde_ähnlich_Termin_vor_Ort 35  4        5.75     1.50
## ENN_28_Zukünftiger_Anspruch                     36  0         NaN       NA
## X                                               37  0         NaN       NA
##                                                   median   trimmed    mad
## ID*                                                 18.0     18.00  13.34
## Gruppe                                               1.0      1.00   0.00
## ENN_00_Datum_RAW*                                   15.0     21.34  13.34
## ENN_00_Datum                                    200714.5 200676.00 289.11
## ENN_1a_Gesprächsteilnehmer                           1.0      1.00   0.00
## ENN_1b_Geschlecht                                    1.0      1.00   0.00
## ENN_1c_Alter                                        31.5     31.50   5.19
## ENN_1d_Schulbildung_Mutter_in_Jahre                 13.0     13.21   3.71
## ENN_1e_beide_Eltern                                  0.0      0.31   0.00
## ENN_2_Endgerät                                        NA       NaN     NA
## ENN_3_Bisheriger_persönlicher_Besuch_im_SPZ          1.0      0.93   0.00
## ENN_4_Zeit_für_Anfahrt                              30.0     27.36  14.83
## ENN_5_Entfernung_km                                 10.5     13.79   8.90
## ENN_6_Urlaub_nehmen_für_Termin                       0.0      0.46   0.00
## ENN_7_Potentielle_Wartezeit                          5.0      7.44   7.41
## ENN_8_Aktive_Rolle_Gesundheit_Kind                   7.0      7.00   0.00
## ENN_9_Qualität_ENN                                   7.0      7.00   0.00
## ENN_10_Zufriedenheit_Video_Audio                     7.0      7.00   0.00
## ENN_11_Pünktlichkeit                                 7.0      6.76   0.00
## ENN_12_Effizienz                                     7.0      7.00   0.00
## ENN_13_Vertrauliche_Weise                            7.0      7.00   0.00
## ENN_14__Sensible_Info                                7.0      7.00   0.00
## ENN_15_Kind_beurteilbar                              7.0      7.00   0.00
## ENN_16_Entspannung_und_Kooperation                   7.0      6.55   0.00
## ENN_17_selber_untersuchen                            6.0      6.00   0.74
## ENN_18_Positionswechsel_Erklärungen                   NA       NaN     NA
## ENN_19_Zeitlicher_Umfang                             7.0      6.92   0.00
```

```
## ENN_20_Atmosphäre                                7.0       6.97   0.00
## ENN_21_gute_Betreuung                            7.0       7.00   0.00
## ENN_27_Zufriedenheit_Insgesamt                   7.0       7.00   0.00
## ENN_22_Häusliche_Umgebung_Sicherer_vor_Erregern   7.0       6.59   0.00
## ENN_23_Kontaktvermeidung_andere_Menschen         6.0       5.69   1.48
## ENN_24_Kontaktvermeidung_medizinisches_Personal   4.0       3.86   2.97
## ENN_25_Sorge_vor_Corona                          6.0       5.59   1.48
## ENN_26_Videosprechstunde_ähnlich_Termin_vor_Ort   6.0       5.75   1.48
## ENN_28_Zukünftiger_Anspruch                       NA        NaN     NA
## X                                                 NA        NaN     NA
##                                                  min    max range  skew
## ID*                                                1     35    34  0.00
## Gruppe                                             1      1     0   NaN
## ENN_00_Datum_RAW*                                  1     55    54  0.63
## ENN_00_Datum                                  200207 210125  9918  4.96
## ENN_1a_Gesprächsteilnehmer                         1      2     1  5.33
## ENN_1b_Geschlecht                                  1      2     1  5.33
## ENN_1c_Alter                                      19     47    28  0.08
## ENN_1d_Schulbildung_Mutter_in_Jahre                8     18    10 -0.02
## ENN_1e_beide_Eltern                                0      1     1  0.63
## ENN_2_Endgerät                                   Inf   -Inf  -Inf    NA
## ENN_3_Bisheriger_persönlicher_Besuch_im_SPZ        0      1     1 -1.95
## ENN_4_Zeit_für_Anfahrt                            10     75    65  1.12
## ENN_5_Entfernung_km                                2     75    73  1.89
## ENN_6_Urlaub_nehmen_für_Termin                     0      5     5  1.84
## ENN_7_Potentielle_Wartezeit                        0     70    70  1.96
## ENN_8_Aktive_Rolle_Gesundheit_Kind                 1     10     9 -2.06
## ENN_9_Qualität_ENN                                 5      7     2 -4.25
## ENN_10_Zufriedenheit_Video_Audio                   7      7     0   NaN
## ENN_11_Pünktlichkeit                               4      7     3 -1.78
## ENN_12_Effizienz                                   6      7     1 -2.83
## ENN_13_Vertrauliche_Weise                          6      7     1 -3.65
## ENN_14__Sensible_Info                              6      7     1 -5.42
## ENN_15_Kind_beurteilbar                            6      7     1 -3.65
## ENN_16_Entspannung_und_Kooperation                 3      7     4 -1.63
## ENN_17_selber_untersuchen                          5      7     2  0.00
## ENN_18_Positionswechsel_Erklärungen              Inf   -Inf  -Inf    NA
## ENN_19_Zeitlicher_Umfang                           3      7     4 -3.25
## ENN_20_Atmosphäre                                  1      7     6 -4.43
## ENN_21_gute_Betreuung                              6      7     1 -3.65
## ENN_27_Zufriedenheit_Insgesamt                     6      7     1 -5.42
## ENN_22_Häusliche_Umgebung_Sicherer_vor_Erregern     3      7     4 -1.55
## ENN_23_Kontaktvermeidung_andere_Menschen           1      7     6 -0.94
## ENN_24_Kontaktvermeidung_medizinisches_Personal     1      7     6 -0.13
## ENN_25_Sorge_vor_Corona                            1      7     6 -0.83
## ENN_26_Videosprechstunde_ähnlich_Termin_vor_Ort     4      7     3 -0.14
## ENN_28_Zukünftiger_Anspruch                      Inf   -Inf  -Inf    NA
## X                                                Inf   -Inf  -Inf    NA
##                                               kurtosis     se
## ID*                                              -1.30   1.73
## Gruppe                                             NaN   0.00
## ENN_00_Datum_RAW*                                -0.92   2.70
## ENN_00_Datum                                     23.98 298.56
## ENN_1a_Gesprächsteilnehmer                       27.17   0.03
## ENN_1b_Geschlecht                                27.17   0.03
## ENN_1c_Alter                                      0.43   1.07
```

```
## ENN_1d_Schulbildung_Mutter_in_Jahre                  -1.01   0.54
## ENN_1e_beide_Eltern                                  -1.64   0.08
## ENN_2_Endgerät                                          NA      NA
## ENN_3_Bisheriger_persönlicher_Besuch_im_SPZ          1.88   0.06
## ENN_4_Zeit_für_Anfahrt                               0.80   2.70
## ENN_5_Entfernung_km                                  3.55   2.80
## ENN_6_Urlaub_nehmen_für_Termin                       2.14   0.24
## ENN_7_Potentielle_Wartezeit                          3.07   3.06
## ENN_8_Aktive_Rolle_Gesundheit_Kind                  11.16   0.22
## ENN_9_Qualität_ENN                                  17.75   0.06
## ENN_10_Zufriedenheit_Video_Audio                      NaN   0.00
## ENN_11_Pünktlichkeit                                 2.49   0.14
## ENN_12_Effizienz                                     6.21   0.05
## ENN_13_Vertrauliche_Weise                           11.68   0.04
## ENN_14__Sensible_Info                               28.17   0.03
## ENN_15_Kind_beurteilbar                             11.68   0.04
## ENN_16_Entspannung_und_Kooperation                   1.62   0.18
## ENN_17_selber_untersuchen                           -1.88   0.41
## ENN_18_Positionswechsel_Erklärungen                    NA      NA
## ENN_19_Zeitlicher_Umfang                            10.89   0.15
## ENN_20_Atmosphäre                                   20.19   0.18
## ENN_21_gute_Betreuung                               11.68   0.04
## ENN_27_Zufriedenheit_Insgesamt                      28.17   0.03
## ENN_22_Häusliche_Umgebung_Sicherer_vor_Erregern      1.31   0.18
## ENN_23_Kontaktvermeidung_andere_Menschen             0.00   0.28
## ENN_24_Kontaktvermeidung_medizinisches_Personal     -1.45   0.38
## ENN_25_Sorge_vor_Corona                             -0.63   0.34
## ENN_26_Videosprechstunde_ähnlich_Termin_vor_Ort     -2.28   0.75
## ENN_28_Zukünftiger_Anspruch                            NA      NA
## X                                                      NA      NA
```

2 normal distribution

2.1 video consulting

```
VideoAppointment <- subset(ENN, ENN$Gruppe == 0)
shapiro.test(VideoAppointment$ENN_8_Aktive_Rolle_Gesundheit_Kind)
```

```
##
##  Shapiro-Wilk normality test
##
## data:  VideoAppointment$ENN_8_Aktive_Rolle_Gesundheit_Kind
## W = 0.21578, p-value = 5.542e-16
```

```
shapiro.test(VideoAppointment$ENN_9_Qualität_ENN)
```

```
##
##  Shapiro-Wilk normality test
##
## data:  VideoAppointment$ENN_9_Qualität_ENN
## W = 0.35277, p-value = 1.467e-14
```

```r
shapiro.test(VideoAppointment$ENN_10_Zufriedenheit_Video_Audio)
```

```
##
##  Shapiro-Wilk normality test
##
## data:  VideoAppointment$ENN_10_Zufriedenheit_Video_Audio
## W = 0.6269, p-value = 6.987e-11
```

```r
shapiro.test(VideoAppointment$ENN_11_Pünktlichkeit)
```

```
##
##  Shapiro-Wilk normality test
##
## data:  VideoAppointment$ENN_11_Pünktlichkeit
## W = 0.54092, p-value = 3.388e-12
```

```r
shapiro.test(VideoAppointment$ENN_12_Effizienz)
```

```
##
##  Shapiro-Wilk normality test
##
## data:  VideoAppointment$ENN_12_Effizienz
## W = 0.39056, p-value = 3.956e-14
```

```r
shapiro.test(VideoAppointment$ENN_13_Vertrauliche_Weise)
```

```
##
##  Shapiro-Wilk normality test
##
## data:  VideoAppointment$ENN_13_Vertrauliche_Weise
## W = 0.23178, p-value = 7.941e-16
```

```r
shapiro.test(VideoAppointment$ENN_14__Sensible_Info)
```

```
##
##  Shapiro-Wilk normality test
##
## data:  VideoAppointment$ENN_14__Sensible_Info
## W = 0.27643, p-value = 2.234e-15
```

```r
shapiro.test(VideoAppointment$ENN_15_Kind_beurteilbar)
```

```
##
##  Shapiro-Wilk normality test
##
## data:  VideoAppointment$ENN_15_Kind_beurteilbar
## W = 0.38518, p-value = 3.426e-14
```

```r
shapiro.test(VideoAppointment$ENN_16_Entspannung_und_Kooperation)
```

```
##
##  Shapiro-Wilk normality test
##
## data:  VideoAppointment$ENN_16_Entspannung_und_Kooperation
## W = 0.52975, p-value = 2.356e-12
```

```r
shapiro.test(VideoAppointment$ENN_17_selber_untersuchen)
```

```
##
##  Shapiro-Wilk normality test
##
## data:  VideoAppointment$ENN_17_selber_untersuchen
## W = 0.38541, p-value = 4.58e-14
```

```r
shapiro.test(VideoAppointment$ENN_18_Positionswechsel_Erklärungen)
```

```
##
##  Shapiro-Wilk normality test
##
## data:  VideoAppointment$ENN_18_Positionswechsel_Erklärungen
## W = 0.3486, p-value = 2.36e-14
```

```r
shapiro.test(VideoAppointment$ENN_19_Zeitlicher_Umfang)
```

```
##
##  Shapiro-Wilk normality test
##
## data:  VideoAppointment$ENN_19_Zeitlicher_Umfang
## W = 0.39456, p-value = 5.844e-14
```

```r
shapiro.test(VideoAppointment$ENN_20_Atmosphäre)
```

```
##
##  Shapiro-Wilk normality test
##
## data:  VideoAppointment$ENN_20_Atmosphäre
## W = 0.27401, p-value = 2.11e-15
```

```r
shapiro.test(VideoAppointment$ENN_21_gute_Betreuung)
```

```
##
##  Shapiro-Wilk normality test
##
## data:  VideoAppointment$ENN_21_gute_Betreuung
## W = 0.23178, p-value = 7.941e-16
```

```r
shapiro.test(VideoAppointment$ENN_22_Häusliche_Umgebung_Sicherer_vor_Erregern)
```

```
##
##  Shapiro-Wilk normality test
##
## data:  VideoAppointment$ENN_22_Häusliche_Umgebung_Sicherer_vor_Erregern
## W = 0.3274, p-value = 8.761e-14
```

```r
shapiro.test(VideoAppointment$ENN_23_Kontaktvermeidung_andere_Menschen)
```

```
##
##  Shapiro-Wilk normality test
##
## data:  VideoAppointment$ENN_23_Kontaktvermeidung_andere_Menschen
## W = 0.82023, p-value = 3.193e-06
```

```r
shapiro.test(VideoAppointment$ENN_24_Kontaktvermeidung_medizinisches_Personal)
```

```
##
##  Shapiro-Wilk normality test
##
## data:  VideoAppointment$ENN_24_Kontaktvermeidung_medizinisches_Personal
## W = 0.82318, p-value = 3.133e-06
```

```r
shapiro.test(VideoAppointment$ENN_25_Sorge_vor_Corona)
```

```
##
##  Shapiro-Wilk normality test
##
## data:  VideoAppointment$ENN_25_Sorge_vor_Corona
## W = 0.71129, p-value = 1.327e-08
```

```r
shapiro.test(VideoAppointment$ENN_26_Videosprechstunde_ähnlich_Termin_vor_Ort)
```

```
##
##  Shapiro-Wilk normality test
##
## data:  VideoAppointment$ENN_26_Videosprechstunde_ähnlich_Termin_vor_Ort
## W = 0.7441, p-value = 1.277e-08
```

```r
shapiro.test(VideoAppointment$ENN_27_Zufriedenheit_Insgesamt)
```

```
##
##  Shapiro-Wilk normality test
##
## data:  VideoAppointment$ENN_27_Zufriedenheit_Insgesamt
## W = 0.32205, p-value = 9.057e-15
```

```r
shapiro.test(VideoAppointment$ENN_28_Zukünftiger_Anspruch)
```

```
##
##  Shapiro-Wilk normality test
##
## data:  VideoAppointment$ENN_28_Zukünftiger_Anspruch
## W = 0.53889, p-value = 4.1e-12
```

Keine Variable ist normalverteilt.

2.2 in person appointment

```r
RealAppointment <- subset(ENN, ENN$Gruppe == 1)

shapiro.test(RealAppointment$ENN_8_Aktive_Rolle_Gesundheit_Kind)
```

```
##
##  Shapiro-Wilk normality test
##
## data:  RealAppointment$ENN_8_Aktive_Rolle_Gesundheit_Kind
## W = 0.43481, p-value = 1.789e-10
```

```r
shapiro.test(RealAppointment$ENN_9_Qualität_ENN)
```

```
##
##  Shapiro-Wilk normality test
##
## data:  RealAppointment$ENN_9_Qualität_ENN
## W = 0.24711, p-value = 3.21e-12
```

```r
shapiro.test(RealAppointment$ENN_11_Pünktlichkeit)
```

```
##
##  Shapiro-Wilk normality test
##
## data:  RealAppointment$ENN_11_Pünktlichkeit
## W = 0.61382, p-value = 7.765e-08
```

```r
shapiro.test(RealAppointment$ENN_12_Effizienz)
```

```
##
##  Shapiro-Wilk normality test
##
## data:  RealAppointment$ENN_12_Effizienz
## W = 0.31688, p-value = 1.305e-11
```

```r
shapiro.test(RealAppointment$ENN_13_Vertrauliche_Weise)
```

```
##
##  Shapiro-Wilk normality test
##
## data:  RealAppointment$ENN_13_Vertrauliche_Weise
## W = 0.24996, p-value = 3.394e-12
```

```
shapiro.test(RealAppointment$ENN_14__Sensible_Info)
```

```
##
##  Shapiro-Wilk normality test
##
## data:  RealAppointment$ENN_14__Sensible_Info
## W = 0.16146, p-value = 6.472e-13
```

```
shapiro.test(RealAppointment$ENN_15_Kind_beurteilbar)
```

```
##
##  Shapiro-Wilk normality test
##
## data:  RealAppointment$ENN_15_Kind_beurteilbar
## W = 0.24996, p-value = 3.394e-12
```

```
shapiro.test(RealAppointment$ENN_16_Entspannung_und_Kooperation)
```

```
##
##  Shapiro-Wilk normality test
##
## data:  RealAppointment$ENN_16_Entspannung_und_Kooperation
## W = 0.65743, p-value = 8.649e-08
```

```
shapiro.test(RealAppointment$ENN_19_Zeitlicher_Umfang)
```

```
##
##  Shapiro-Wilk normality test
##
## data:  RealAppointment$ENN_19_Zeitlicher_Umfang
## W = 0.41274, p-value = 4.839e-10
```

```
shapiro.test(RealAppointment$ENN_20_Atmosphäre)
```

```
##
##  Shapiro-Wilk normality test
##
## data:  RealAppointment$ENN_20_Atmosphäre
## W = 0.29854, p-value = 8.945e-12
```

```
shapiro.test(RealAppointment$ENN_21_gute_Betreuung)
```

```
##
##  Shapiro-Wilk normality test
##
## data:  RealAppointment$ENN_21_gute_Betreuung
## W = 0.24996, p-value = 3.394e-12
```

```
shapiro.test(RealAppointment$ENN_22_Häusliche_Umgebung_Sicherer_vor_Erregern)
```

```
##
##  Shapiro-Wilk normality test
##
## data:  RealAppointment$ENN_22_Häusliche_Umgebung_Sicherer_vor_Erregern
## W = 0.64463, p-value = 5.702e-08
```

```
shapiro.test(RealAppointment$ENN_23_Kontaktvermeidung_andere_Menschen)
```

```
##
##  Shapiro-Wilk normality test
##
## data:  RealAppointment$ENN_23_Kontaktvermeidung_andere_Menschen
## W = 0.84576, p-value = 0.000185
```

```
shapiro.test(RealAppointment$ENN_24_Kontaktvermeidung_medizinisches_Personal)
```

```
##
##  Shapiro-Wilk normality test
##
## data:  RealAppointment$ENN_24_Kontaktvermeidung_medizinisches_Personal
## W = 0.85388, p-value = 0.0002827
```

```
shapiro.test(RealAppointment$ENN_25_Sorge_vor_Corona)
```

```
##
##  Shapiro-Wilk normality test
##
## data:  RealAppointment$ENN_25_Sorge_vor_Corona
## W = 0.79754, p-value = 1.827e-05
```

```
shapiro.test(RealAppointment$ENN_27_Zufriedenheit_Insgesamt)
```

```
##
##  Shapiro-Wilk normality test
##
## data:  RealAppointment$ENN_27_Zufriedenheit_Insgesamt
## W = 0.16146, p-value = 6.472e-13
```

No variable is normally distributed.

3 GROUP DIFFERENCES: Calculated group differences between a video consultation and an outpatient appointment.

3.1 Mann-Whitney U test

3.2 First significant difference

```
describeBy(ENN$ENN_1c_Alter, ENN$Gruppe)
```

```
##
##  Descriptive statistics by group
## group: 0
##    vars  n  mean   sd median trimmed  mad min max range  skew kurtosis   se
## X1    1 55 33.45 5.11     35   33.84 4.45  21  42    21 -0.68    -0.19 0.69
## ------------------------------------------------------------
## group: 1
##    vars  n mean   sd median trimmed  mad min max range skew kurtosis   se
## X1    1 30 31.4 5.86   31.5    31.5 5.19  19  47    28 0.08     0.43 1.07
```

```
boxplot(ENN_1c_Alter ~ Gruppe, data=ENN)
```

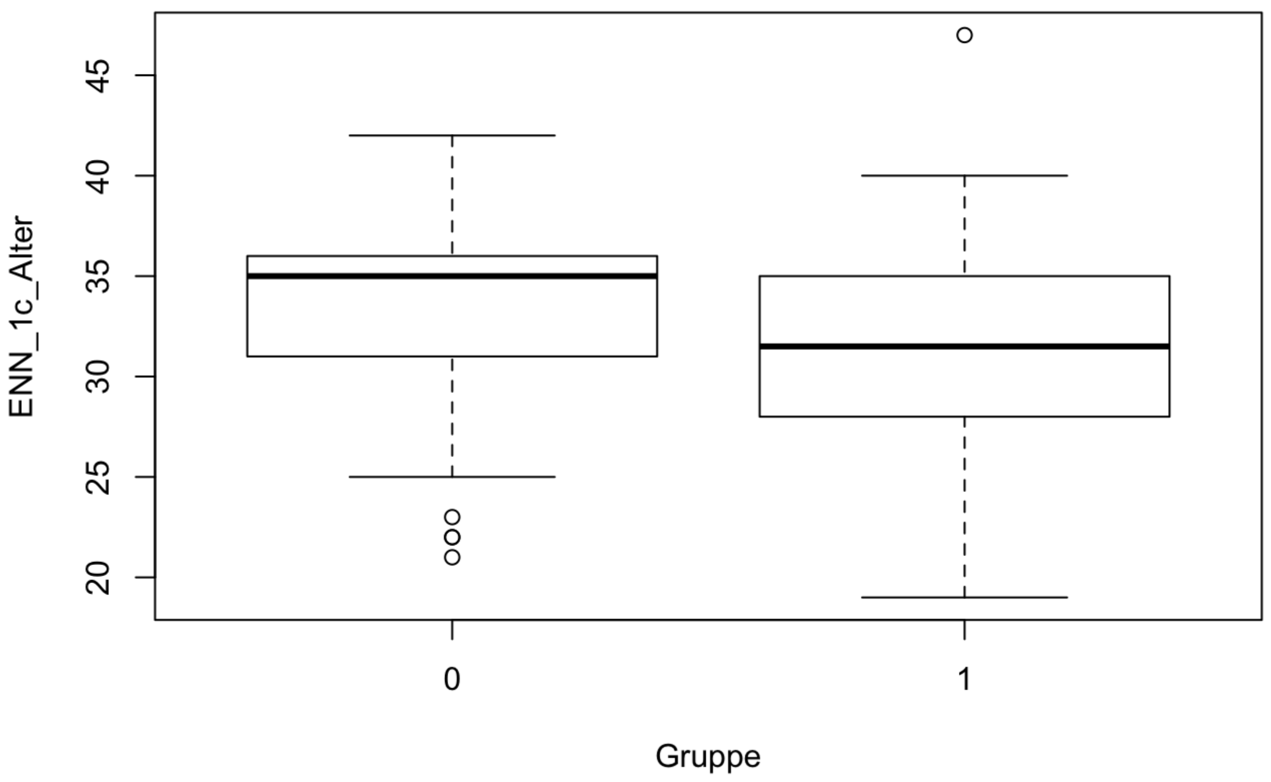

```
wilcox.test(ENN_1c_Alter ~ Gruppe, data=ENN, exact=FALSE, correct = FALSE, conf.int=T
RUE)
```

```
##
##  Wilcoxon rank sum test
##
## data:  ENN_1c_Alter by Gruppe
## W = 1043, p-value = 0.04413
## alternative hypothesis: true location shift is not equal to 0
## 95 percent confidence interval:
##  1.210346e-05 4.999981e+00
## sample estimates:
## difference in location
##               2.999965
```

There is a significant difference between the groups.

Calculate effect size r (effect size according to Cohen: 0.1-0.29=small; 0.3-0.40=medium; > 0.5=strong)

```
z <- qnorm(0.04413)
r <- z/sqrt(93)
r
```

```
## [1] -0.1767638
```

There is a small effect between the groups.

```
ggplot(data=ENN, mapping=aes(x=Gruppe, y=ENN_1c_Alter)) +
  stat_summary(fun.data=mean_sdl, geom="bar") +
  theme_classic()+
  labs(title="Differences in age between video consultation and real appointment grou
ps", x="Gruppe", y="Alter in Jahren")
```

```
## Warning: Removed 8 rows containing non-finite values (stat_summary).
```

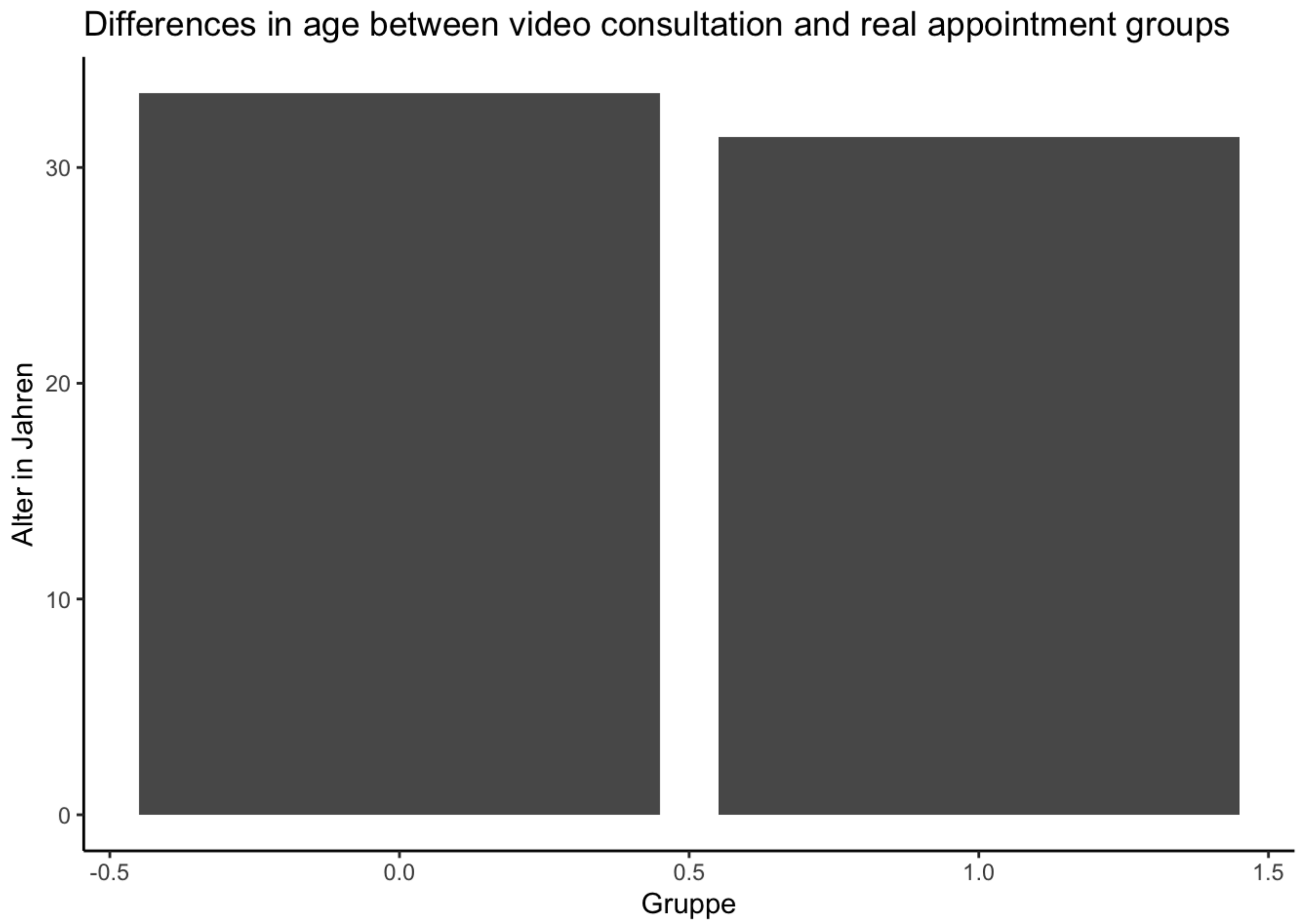

describeBy(ENN$ENN_1 cc_A lter, ENN$Gruppe) boxplot(ENN_1cc_Alter ~ Gruppe, data=ENN) wilcox.test(ENN_1cc_Alter ~ Gruppe, data=ENN, exact=FALSE, correct = FALSE, conf.int=TRUE) ### There is no significant difference between the groups.

describeBy(ENN$ENN_1 d_S chulbildung_M utter_i n_J ahre, ENN$Gruppe) boxplot(ENN_1d_Schulbildung_Mutter_in_Jahre ~ Gruppe, data=ENN) wilcox.test(ENN_1d_Schulbildung_Mutter_in_Jahre ~ Gruppe, data=ENN, exact=FALSE, correct = FALSE, conf.int=TRUE) ### There is no significant difference between the groups.

describeBy(ENN$ENN_1 dd_S chulbildung_V ater_i n_J ahren, ENN$Gruppe) boxplot(ENN_1dd_Schulbildung_Vater_in_Jahren ~ Gruppe, data=ENN) wilcox.test(ENN_1dd_Schulbildung_Vater_in_Jahren ~ Gruppe, data=ENN, exact=FALSE, correct = FALSE, conf.int=TRUE) ### There is no significant difference between the groups.

describeBy(ENN ENN_6_Urlaub_nehmen_für_Termin, ENN Gruppe) boxplot(ENN_6_Urlaub_nehmen_für_Termin~ Gruppe, data=ENN) wilcox.test(ENN_6_Urlaub_nehmen_für_Termin ~ Gruppe, data=ENN, exact=FALSE, correct = FALSE, conf.int=TRUE) ### There is no significant difference between the groups.

3.3 Second significant difference

```
describeBy(ENN$ENN_7_Potentielle_Wartezeit, ENN$Gruppe)
```

```
##
##  Descriptive statistics by group
## group: 0
##    vars  n  mean    sd median trimmed   mad min max range skew kurtosis   se
## X1    1 57 23.58 27.75     15   18.66 14.83   1 180   179 3.45    15.56 3.68
## ------------------------------------------------------------
## group: 1
##    vars  n  mean   sd median trimmed  mad min max range skew kurtosis   se
## X1    1 33 11.39 17.6      5    7.44 7.41   0  70    70 1.96     3.07 3.06
```

```
boxplot(ENN_7_Potentielle_Wartezeit ~ Gruppe, data=ENN)
```

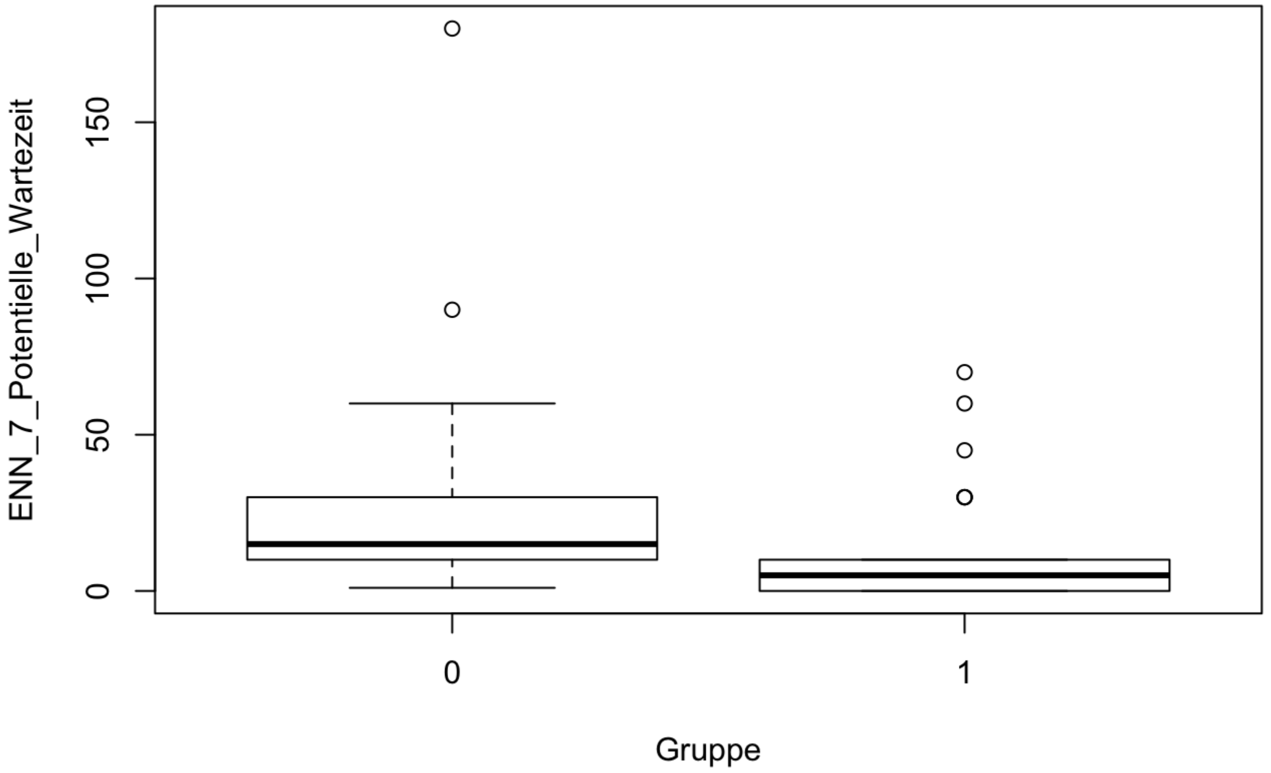


```
wilcox.test(ENN_7_Potentielle_Wartezeit ~ Gruppe, data=ENN, exact=FALSE, correct = FA
LSE, conf.int=TRUE)
```

```
##
##  Wilcoxon rank sum test
##
## data:  ENN_7_Potentielle_Wartezeit by Gruppe
## W = 1387.5, p-value = 0.0001541
## alternative hypothesis: true location shift is not equal to 0
## 95 percent confidence interval:
##   4.999957 14.999983
## sample estimates:
## difference in location
##               9.999962
```

There is a significant difference between the groups.

Calculate effect size r (effect size according to Cohen: 0.1-0.29=small; 0.3-0.40=medium; > 0.5=strong)

```
z <- qnorm(0.0002289)
r <- z/sqrt(93)
r
```

```
## [1] -0.3633795
```

There is a medium effect between groups.

```
ggplot(data=ENN, mapping=aes(x=Gruppe, y=ENN_7_Potentielle_Wartezeit)) +
  stat_summary(fun.data=mean_sdl, geom="bar") +
  theme_classic()+
  labs(title="Differences in potential waiting time between video consultation and re
al appointment groups.", x="Gruppe", y="Wartezeit in Minuten")
```

```
## Warning: Removed 3 rows containing non-finite values (stat_summary).
```

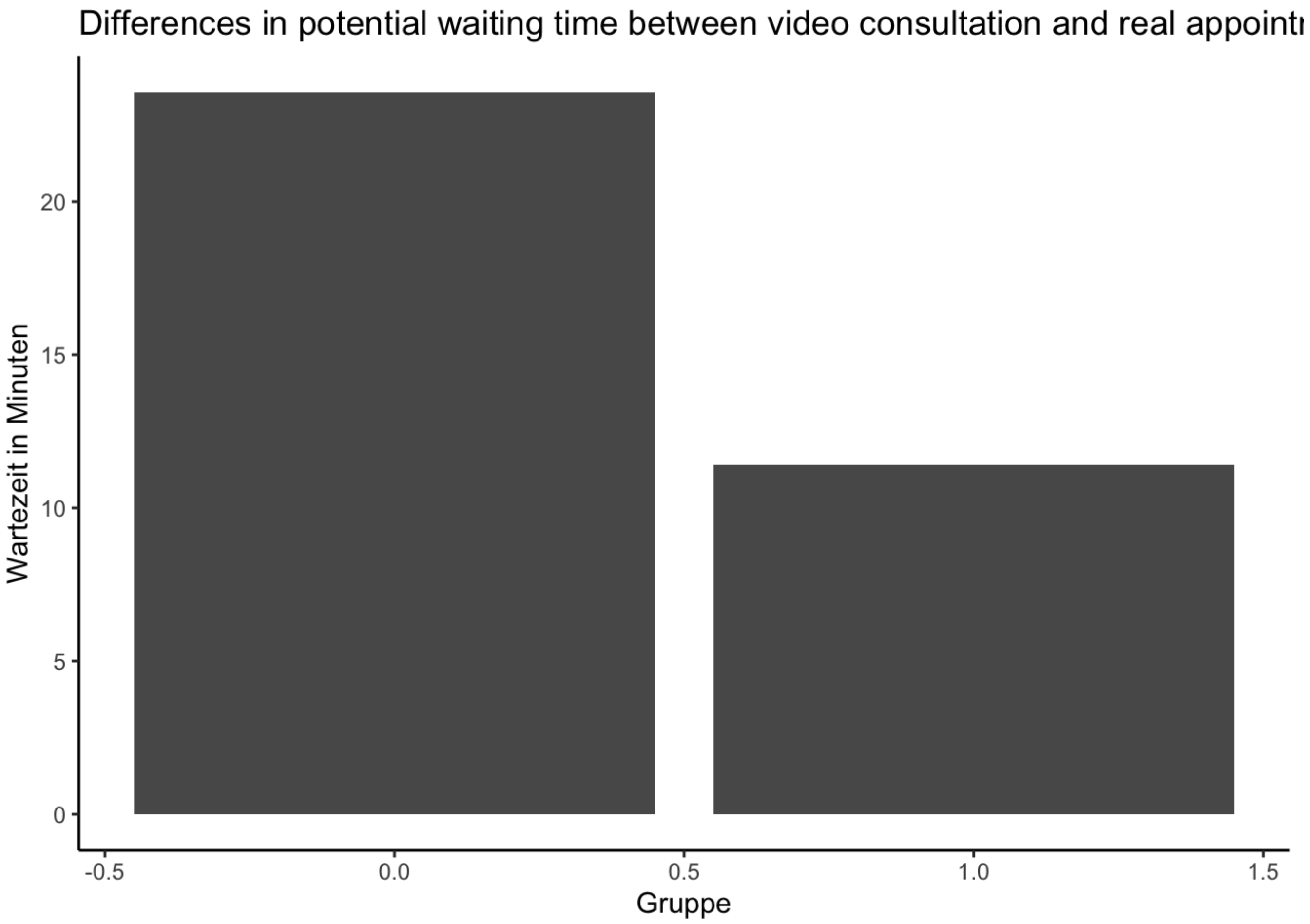

describeBy(ENN ENN_8_Aktive_Rolle_Gesundheit_Kind, ENN Gruppe)
boxplot(ENN_8_Aktive_Rolle_Gesundheit_Kind ~ Gruppe, data=ENN)
wilcox.test(ENN_8_Aktive_Rolle_Gesundheit_Kind~ Gruppe, data=ENN, exact=FALSE, correct = FALSE, conf.int=TRUE) ### There is no significant difference between the groups.

describeBy(ENN ENN_9_Qualität_ENN, ENN Gruppe) boxplot(ENN_9_Qualität_ENN ~ Gruppe, data=ENN) wilcox.test(ENN_9_Qualität_ENN ~ Gruppe, data=ENN, exact=FALSE, correct = FALSE, conf.int=TRUE) ### There is no significant difference between the groups.

describeBy(ENN$ENN_11_Pünktlichkeit, ENN$Gruppe) boxplot(ENN_11_Pünktlichkeit ~ Gruppe, data=ENN) wilcox.test(ENN_11_Pünktlichkeit ~ Gruppe, data=ENN, exact=FALSE, correct = FALSE, conf.int=TRUE) ### There is no significant difference between the groups.

describeBy(ENN$ENN_12_Effizienz, ENN$Gruppe) boxplot(ENN_12_Effizienz ~ Gruppe, data=ENN) wilcox.test(ENN_12_Effizienz ~ Gruppe, data=ENN, exact=FALSE, correct = FALSE, conf.int=TRUE) ### There is no significant difference between the groups.

describeBy(ENN$ENN_13_Vertrauliche_Weise, ENN$Gruppe) boxplot(ENN_13_Vertrauliche_Weise ~ Gruppe, data=ENN) wilcox.test(ENN_13_Vertrauliche_Weise ~ Gruppe, data=ENN, exact=FALSE, correct = FALSE, conf.int=TRUE) ### There is no significant difference between the groups.

describeBy(ENN ENN_14__Sensible_Info, ENN Gruppe) boxplot(ENN_14__Sensible_Info ~ Gruppe, data=ENN) wilcox.test(ENN_14__Sensible_Info ~ Gruppe, data=ENN, exact=FALSE, correct = FALSE, conf.int=TRUE) ### There is no significant difference between the groups.

describeBy(ENN$ENN_15_Kind_beurteilbar, ENN$Gruppe) boxplot(ENN_15_Kind_beurteilbar ~ Gruppe, data=ENN) wilcox.test(ENN_15_Kind_beurteilbar ~ Gruppe, data=ENN, exact=FALSE, correct = FALSE, conf.int=TRUE) ### There is no significant difference between the groups.

describeBy(ENN$ENN_16_Entspannung_und_Kooperation, ENN$Gruppe) boxplot(ENN_16_Entspannung_und_Kooperation ~ Gruppe, data=ENN) wilcox.test(ENN_16_Entspannung_und_Kooperation ~ Gruppe, data=ENN, exact=FALSE, correct = FALSE, conf.int=TRUE) ### There is no significant difference between the groups.

describeBy(ENN$ENN_19_Zeitlicher_Umfang, ENN$Gruppe) boxplot(ENN_19_Zeitlicher_Umfang ~ Gruppe, data=ENN) wilcox.test(ENN_19_Zeitlicher_Umfang ~ Gruppe, data=ENN, exact=FALSE, correct = FALSE, conf.int=TRUE) ### There is no significant difference between the groups.

describeBy(ENN$ENN_20_Atmosphäre, ENN$Gruppe) boxplot(ENN_20_Atmosphäre ~ Gruppe, data=ENN) wilcox.test(ENN_20_Atmosphäre ~ Gruppe, data=ENN, exact=FALSE, correct = FALSE, conf.int=TRUE) ### There is no significant difference between the groups.

describeBy(ENN$ENN_21_gute_Betreuung, ENN$Gruppe) boxplot(ENN_21_gute_Betreuung ~ Gruppe, data=ENN) wilcox.test(ENN_21_gute_Betreuung ~ Gruppe, data=ENN, exact=FALSE, correct = FALSE, conf.int=TRUE) ### There is no significant difference between the groups.

3.4 Third significant difference

```
describeBy(ENN$ENN_22_Häusliche_Umgebung_Sicherer_vor_Erregern, ENN$Gruppe)
```

```
##
##  Descriptive statistics by group
## group: 0
##    vars  n mean   sd median trimmed mad min max range  skew kurtosis   se
## X1    1 50 6.78 0.76      7       7   0   3   7     4 -3.66    13.15 0.11
## ------------------------------------------------------------
## group: 1
##    vars  n mean   sd median trimmed mad min max range  skew kurtosis   se
## X1    1 35 6.37 1.09      7    6.59   0   3   7     4 -1.55     1.31 0.18
```

```
boxplot(ENN_22_Häusliche_Umgebung_Sicherer_vor_Erregern ~ Gruppe, data=ENN)
```

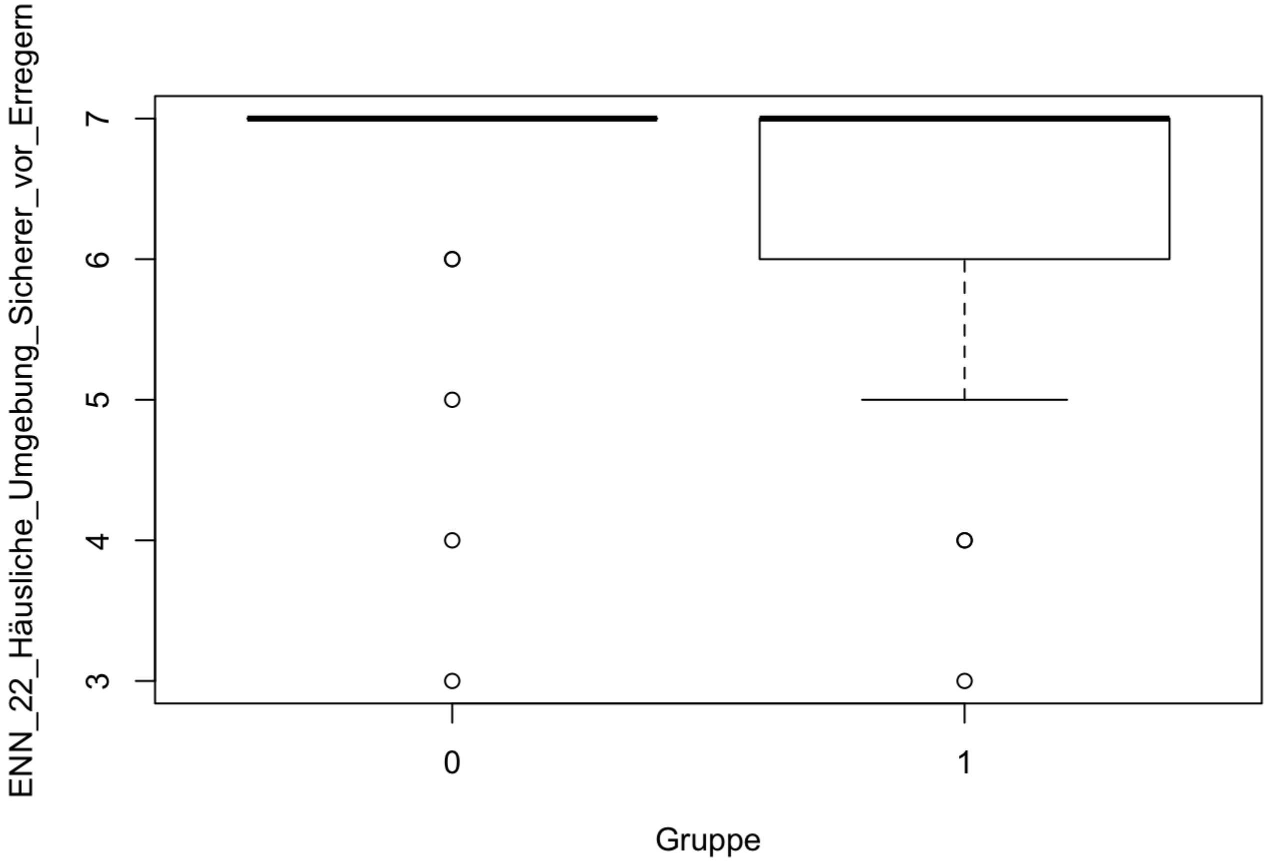


```
wilcox.test(ENN_22_Häusliche_Umgebung_Sicherer_vor_Erregern ~ Gruppe, data=ENN, exact
=FALSE, correct = FALSE, conf.int=TRUE)
```

```
##
##  Wilcoxon rank sum test
##
## data:  ENN_22_Häusliche_Umgebung_Sicherer_vor_Erregern by Gruppe
## W = 1060.5, p-value = 0.01509
## alternative hypothesis: true location shift is not equal to 0
## 95 percent confidence interval:
##  -4.846475e-05  7.294688e-05
## sample estimates:
## difference in location
##           4.095156e-05
```

There is a significant difference between the groups.

Calculate effect size r (effect size according to Cohen: 0.1-0.29=small; 0.3-0.40=medium; > 0.5=strong)

```
z <- qnorm(0.006462)
r <- z/sqrt(93)
r
```

```
## [1] -0.2577713
```

There is a small effect between the groups.

```
ggplot(data=ENN, mapping=aes(x=Gruppe, y=ENN_22_Häusliche_Umgebung_Sicherer_vor_Erreg
ern)) +
  stat_summary(fun.data=mean_sdl, geom="bar") +
  theme_classic()+
  labs(title="Differences in perceived safety from potential pathogens in own home en
vironment between video consultation and real appointment groups.", x="Gruppe", y="Si
cherheit")
```

```
## Warning: Removed 8 rows containing non-finite values (stat_summary).
```

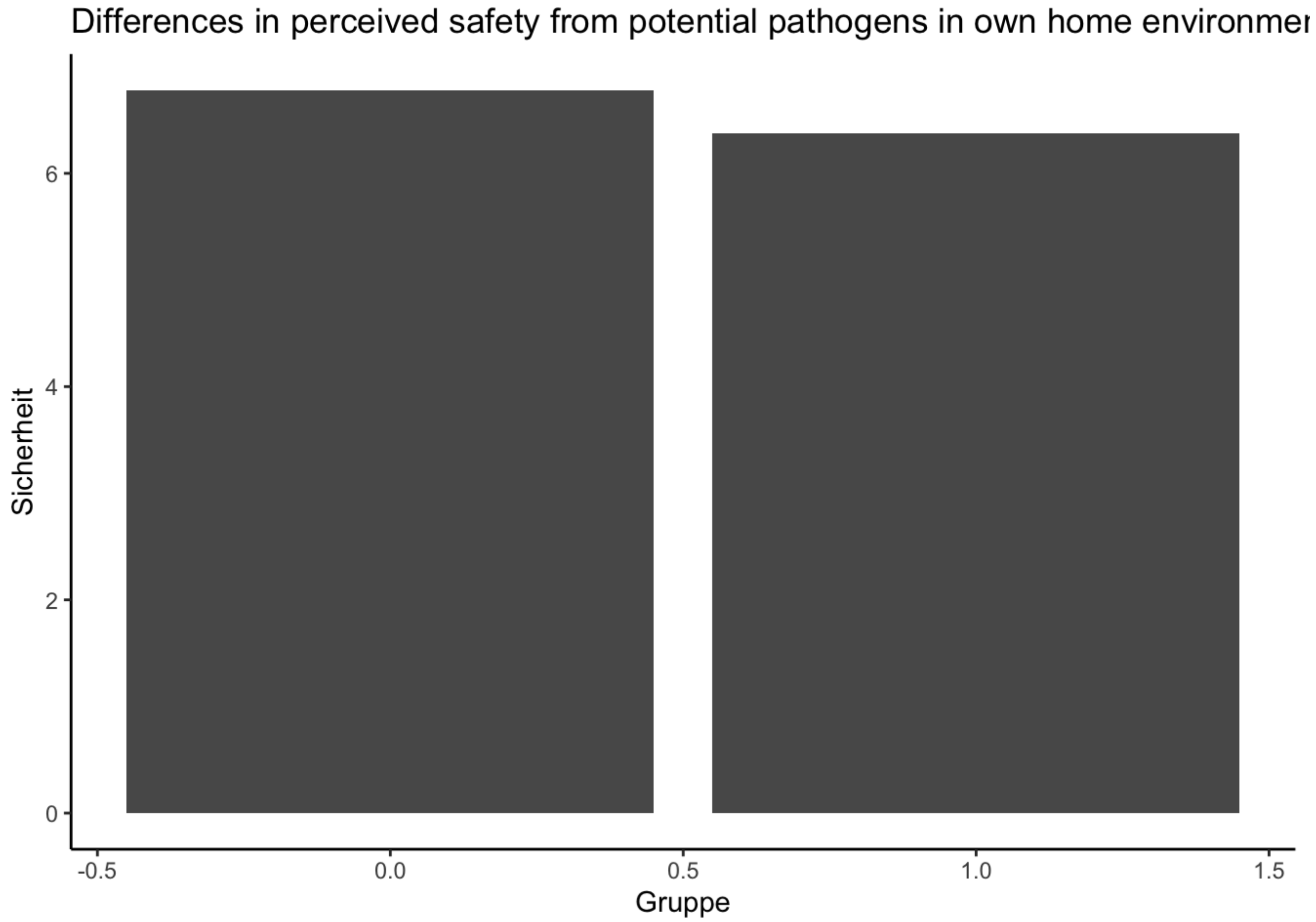

describeBy(ENN$ENN_2 3_K ontaktvermeidung_a ndere_M enschen, ENN$Gruppe)
boxplot(ENN_23_Kontaktvermeidung_andere_Menschen ~ Gruppe, data=ENN)
wilcox.test(ENN_23_Kontaktvermeidung_andere_Menschen ~ Gruppe, data=ENN, exact=FALSE, correct = FALSE, conf.int=TRUE)

3.5 Fourth significant difference

```
describeBy(ENN$ENN_24_Kontaktvermeidung_medizinisches_Personal, ENN$Gruppe)
```

```
##
##  Descriptive statistics by group
## group: 0
##    vars  n mean   sd median trimmed  mad min max range  skew kurtosis   se
## X1    1 50 4.82 2.26      5    5.03 2.97   1   7     6 -0.62    -1.11 0.32
## ------------------------------------------------------------
## group: 1
##    vars  n mean   sd median trimmed  mad min max range  skew kurtosis   se
## X1    1 35 3.89 2.25      4    3.86 2.97   1   7     6 -0.13    -1.45 0.38
```

```
boxplot(ENN_24_Kontaktvermeidung_medizinisches_Personal ~ Gruppe, data=ENN)
```

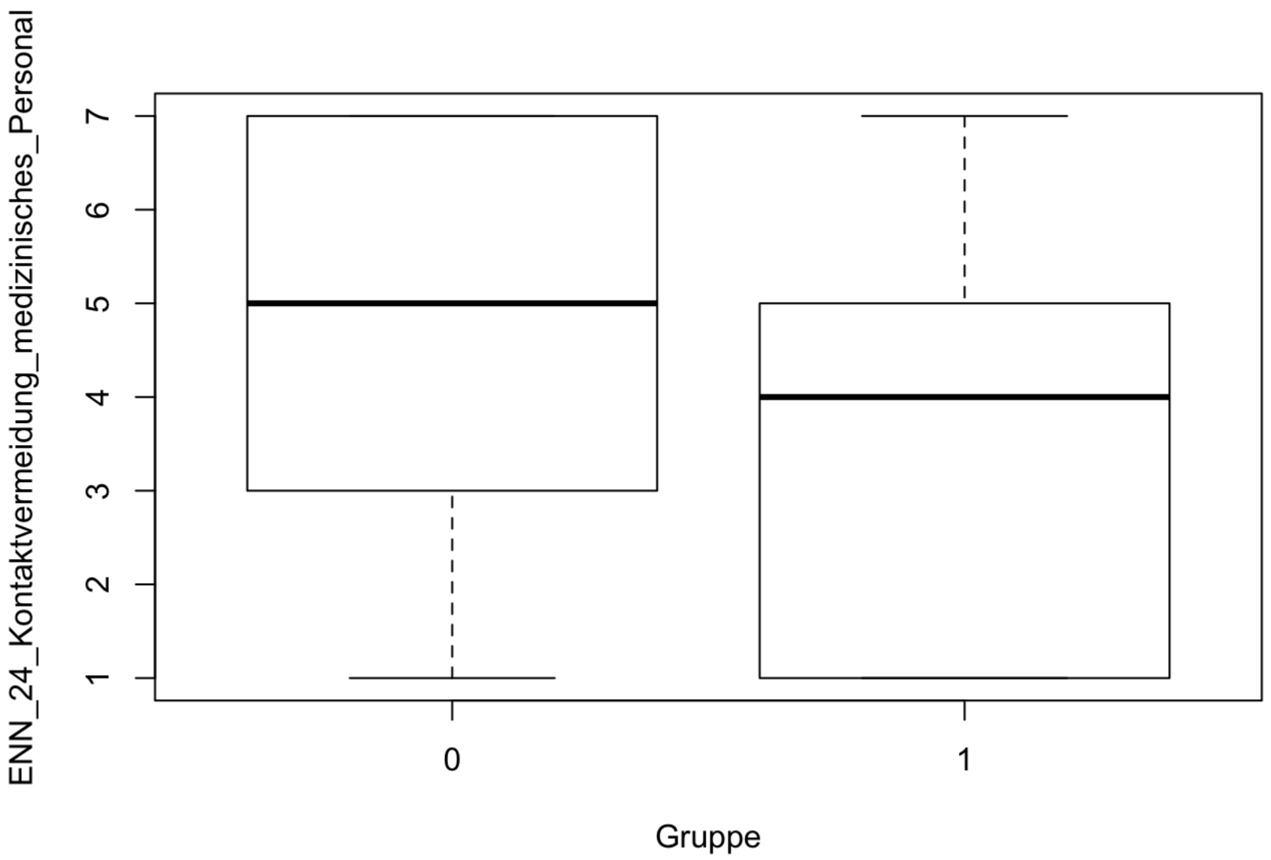


```
wilcox.test(ENN_24_Kontaktvermeidung_medizinisches_Personal ~ Gruppe, data=ENN, exact
=FALSE, correct = FALSE, conf.int=TRUE)
```

```
##
##  Wilcoxon rank sum test
##
## data:  ENN_24_Kontaktvermeidung_medizinisches_Personal by Gruppe
## W = 1094.5, p-value = 0.04481
## alternative hypothesis: true location shift is not equal to 0
## 95 percent confidence interval:
##  1.889837e-05 2.000006e+00
## sample estimates:
## difference in location
##              0.9999555
```

There is a significant difference between the groups.

Calculate effect size r (effect size according to Cohen: 0.1-0.29=small; 0.3-0.40=medium; > 0.5=strong)

```
z <- qnorm(0.04284)
r <- z/sqrt(93)
r
```

```
## [1] -0.1782146
```

There is a small effect between the groups

```
ggplot(data=ENN, mapping=aes(x=Gruppe, y=ENN_24_Kontaktvermeidung_medizinisches_Perso
nal)) +
  stat_summary(fun.data=mean_sdl, geom="bar") +
  theme_classic()+
  labs(title="Differences in medical staff contact avoidance between video consultati
on and real appointment groups.", x="Gruppe", y="Kontaktvermeidung")
```

```
## Warning: Removed 8 rows containing non-finite values (stat_summary).
```

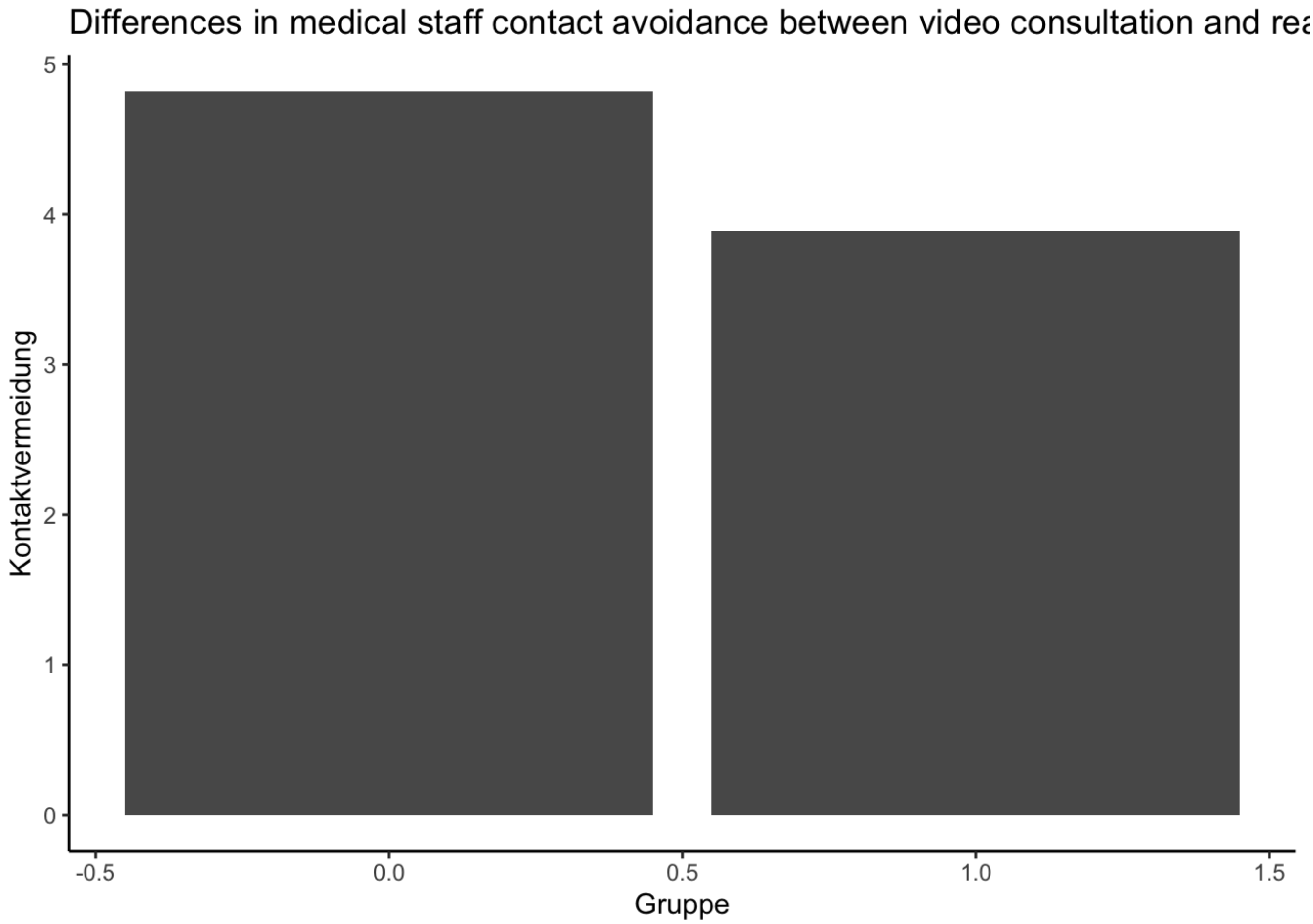

describeBy(ENN$ENN_{2}5_{S}orge_{v}or_{C}orona, ENN$Gruppe) boxplot(ENN_25_Sorge_vor_Corona ~ Gruppe, data=ENN) wilcox.test(ENN_25_Sorge_vor_Corona ~ Gruppe, data=ENN, exact=FALSE, correct = FALSE, conf.int=TRUE) ### There is no significant difference between the groups.

4 LOCKDOWN

4.1 Distribution over time of interviews conducted.

```
Lockdown <- rep(0, nrow(ENN))

Lockdown[ENN$ENN_00_Datum<=200506] <- 1
Lockdown[ENN$ENN_00_Datum>=201028 & ENN$ENN_00_Datum<=201125] <- 2
Lockdown[ENN$ENN_00_Datum>=201213 & ENN$ENN_00_Datum<= 210125] <- 3

ENN$Lockdown=Lockdown

kreuztabelle <- xtabs(~ ENN$Lockdown + ENN$Gruppe)
kreuztabelle
```

```
##              ENN$Gruppe
## ENN$Lockdown  0  1
##             0 37 26
##             1 12  6
##             2  9  0
##             3  0  3
```

```
counts <- table(ENN$Gruppe, ENN$Lockdown)
barplot(counts, main="Frequencies of interviews during the 3 lockdowns",
  xlab="Lockdown", col=c("darkblue","red"),
  legend = rownames(counts), beside=TRUE)
```

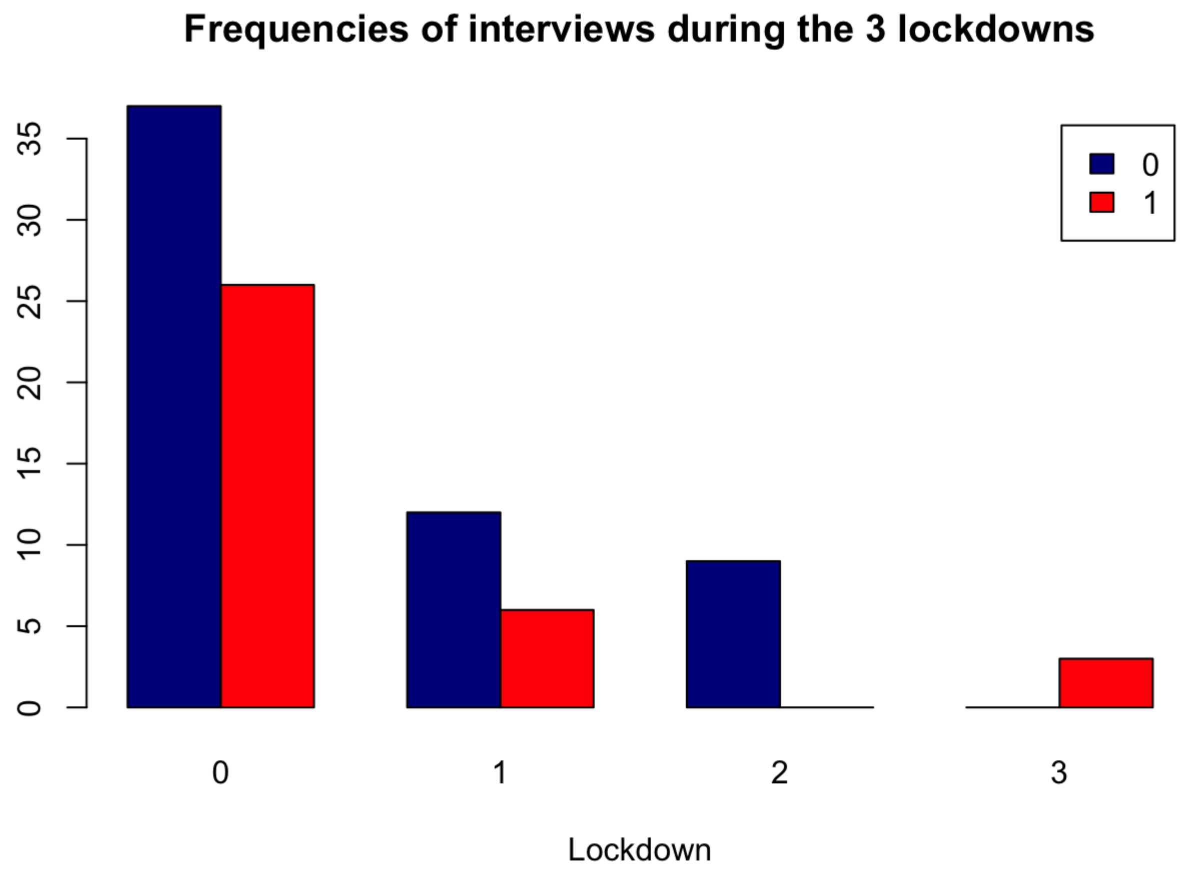

Video consultation (group=0) Real appointment (group=1)

0. interviews between the performed lockdowns
1. lockdown was everything before 06.50.2020
2. lockdown was from 10/28/2020 to 11/25/2020
3. lockdown was from 12/13/2020 to 01/25/2021

4.2 Calculation of the differences between the lockdowns

```
kruskal.test(ENN$Lockdown, ENN$ENN_22_Häusliche_Umgebung_Sicherer_vor_Erregern)
```

```
##
##  Kruskal-Wallis rank sum test
##
## data:  ENN$Lockdown and ENN$ENN_22_Häusliche_Umgebung_Sicherer_vor_Erregern
## Kruskal-Wallis chi-squared = 2.7565, df = 4, p-value = 0.5994
```

```
kruskal.test(ENN$Lockdown, ENN$ENN_23_Kontaktvermeidung_andere_Menschen)
```

```
##
##  Kruskal-Wallis rank sum test
##
## data:  ENN$Lockdown and ENN$ENN_23_Kontaktvermeidung_andere_Menschen
## Kruskal-Wallis chi-squared = 2.0334, df = 6, p-value = 0.9166
```

```
kruskal.test(ENN$Lockdown, ENN$ENN_24_Kontaktvermeidung_medizinisches_Personal)
```

```
##
##  Kruskal-Wallis rank sum test
##
## data:  ENN$Lockdown and ENN$ENN_24_Kontaktvermeidung_medizinisches_Personal
## Kruskal-Wallis chi-squared = 12.218, df = 6, p-value = 0.05727
```

```
kruskal.test(ENN$Lockdown, ENN$ENN_25_Sorge_vor_Corona)
```

```
##
##  Kruskal-Wallis rank sum test
##
## data:  ENN$Lockdown and ENN$ENN_25_Sorge_vor_Corona
## Kruskal-Wallis chi-squared = 7.596, df = 6, p-value = 0.2692
```

There is no significant difference between the different lockdowns in terms of safety from corona in one's home environment, avoidance of contact with other people and medical staff, as well as concern about corona for themself and their child.

5 EXPLORATORY DATA ANALYSIS

5.1 Spearman rank correlations

```
cor.test(ENN$Lockdown, ENN$ENN_27_Zufriedenheit_Insgesamt, method="spearman")
```

```
## Warning in cor.test.default(ENN$Lockdown, ENN$ENN_27_Zufriedenheit_Insgesamt, :
## Cannot compute exact p-value with ties
```

```
##
##  Spearman's rank correlation rho
##
## data:  ENN$Lockdown and ENN$ENN_27_Zufriedenheit_Insgesamt
## S = 121041, p-value = 0.5243
## alternative hypothesis: true rho is not equal to 0
## sample estimates:
##        rho
## 0.06723347
```

6 Figure

```r
## Figure 1

RHI <- read.table("figure_1.txt", header=TRUE, sep="\t")

#png("figure 1.png")#, width = 700, height = 800, res = 300)
dodge <- position_dodge(.5)
plot1 <- ggplot(data=RHI, aes(x=Item, y=M, fill=Group))+
  geom_bar(stat="identity", width=.25, position=dodge)+
  #scale_fill_manual(values=c("#1874CD", "#A2CD5A"))+
  geom_errorbar(aes(ymin=M, ymax=M+SD), width=.05, position=dodge)+
  geom_signif(
    y_position = c(42), xmin = c(0.8), xmax = c(1.2),
    annotation = c("*"), tip_length = 0)+
  ylab("Age in Years")+
  #xlab("Item")+
  ggtitle("a)")+
  theme_minimal(base_size=10)+
  ylim(0, 45)+
  theme(legend.position="none")
#guides(fill=FALSE)

#dev.off()

## figure 2

RHI <- read.table("figure_2.txt", header=TRUE, sep="\t")

#png("figure 2.png")#, width = 700, height = 800, res = 300)
dodge <- position_dodge(.5)
plot2 <- ggplot(data=RHI, aes(x=Item, y=M, fill=Group))+
  geom_bar(stat="identity", width=.25, position=dodge)+
  #scale_fill_manual(values=c("#1874CD", "#A2CD5A"))+
  geom_errorbar(aes(ymin=M, ymax=M+SD), width=.05, position=dodge)+
  geom_signif(
    y_position = c(56), xmin = c(0.8), xmax = c(1.2),
    annotation = c("*"), tip_length = 0)+
  #geom_signif(comparisons=list(c("Group", "waiting time")), annotations="***",y_posi
tion = 30, tip_length = 0, vjust=0.4)+
  ylab("Waiting Time in Minutes")+
  #xlab("Item")+
  ggtitle("b)")+
  theme_minimal(base_size=10)+
  ylim(0, 60)+
  theme(legend.position="none")
#guides(fill=FALSE)

#dev.off()


## figure 3

RHI <- read.table("figure_3.txt", header=TRUE, sep="\t")

#png("figure 3.png")#, width = 1600, height = 800, res = 300)
dodge <- position_dodge(.5)
```

```
plot3 <- ggplot(data=RHI, aes(x=Items, y=M, fill=Group))+
  geom_bar(stat="identity", width=.5, position=dodge)+
  #scale_fill_manual(values=c("#1874CD", "#A2CD5A"))+
  geom_errorbar(aes(ymin=M, ymax=M+SD), width=.05, position=dodge)+
  geom_signif(
    y_position = c(8, 8), xmin = c(0.8, 1.8), xmax = c(1.2, 2.2),
    annotation = c("*", "*"), tip_length = 0)+
  ylab("Level of Consent")+
  ggtitle("c)")+
  #xlab("Items")+
  theme_minimal(base_size=10)+
  ylim(0, 10)+
  theme(legend.position="right")
#guides(fill=FALSE)

#dev.off()

#All plots together

#png("figure_together.png", width = 3500, height = 800, res = 300)
dodge <- position_dodge(.5)
plot1 + plot2 + plot3
```

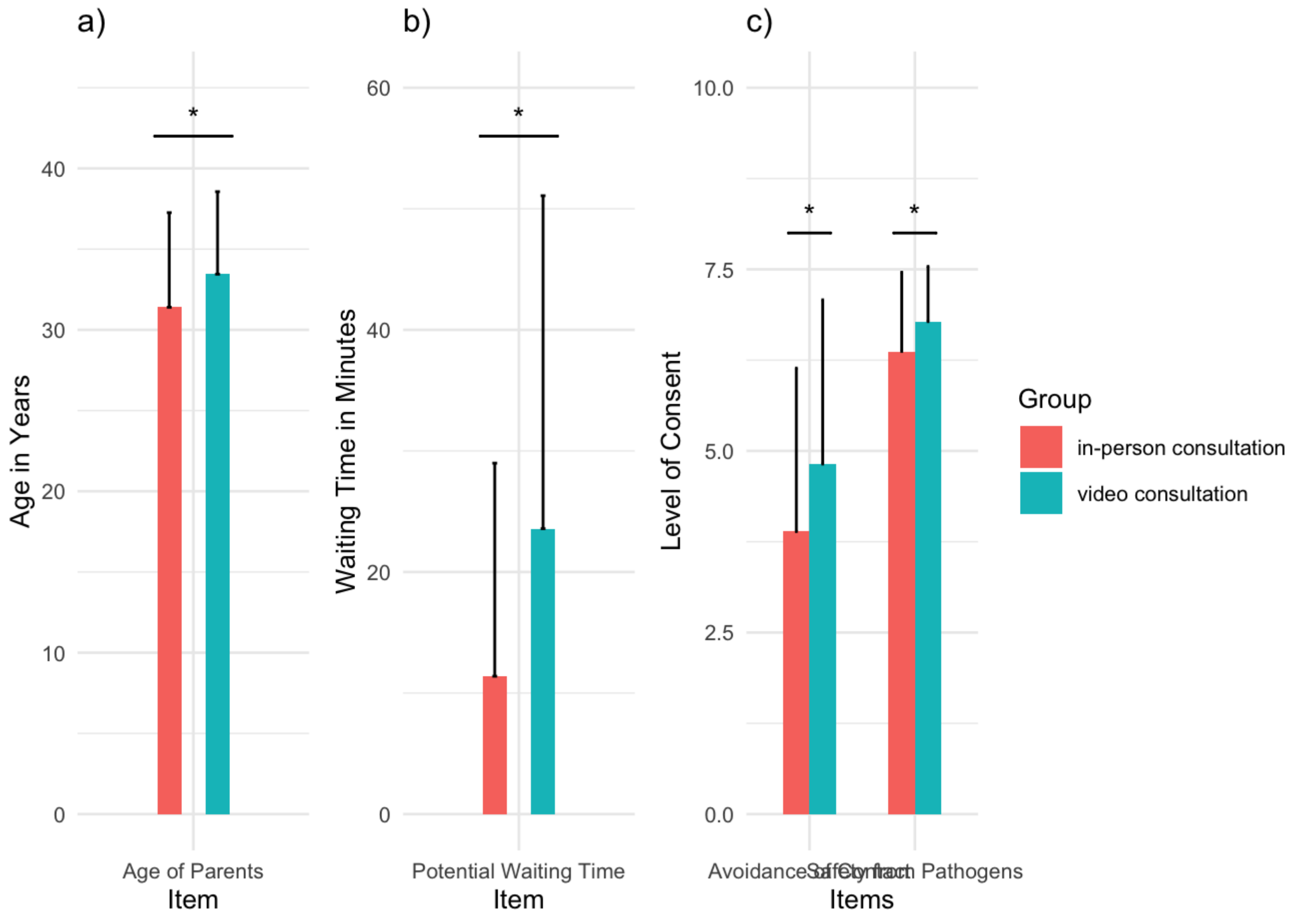


```
#dev.off()
```
